# Tsr4 and Nap1, two novel members of the ribosomal protein chaperOME

**DOI:** 10.1093/nar/gkz317

**Published:** 2019-05-07

**Authors:** Ingrid Rössler, Julia Embacher, Benjamin Pillet, Guillaume Murat, Laura Liesinger, Jutta Hafner, Julia Judith Unterluggauer, Ruth Birner-Gruenberger, Dieter Kressler, Brigitte Pertschy

**Affiliations:** 1 Institute of Molecular Biosciences, University of Graz, Humboldtstrasse 50, 8010 Graz, Austria; 2 BioTechMed-Graz, Graz, Austria; 3 Unit of Biochemistry, Department of Biology, University of Fribourg, Chemin du Musée 10, 1700 Fribourg, Switzerland; 4 Gottfried Schatz Research Center, Medical University of Graz, Graz, Austria; 5 Omics Center Graz, BioTechMed-Graz, Graz, Austria

## Abstract

Dedicated chaperones protect newly synthesized ribosomal proteins (r-proteins) from aggregation and accompany them on their way to assembly into nascent ribosomes. Currently, only nine of the ∼80 eukaryotic r-proteins are known to be guarded by such chaperones. In search of new dedicated r-protein chaperones, we performed a tandem-affinity purification based screen and looked for factors co-enriched with individual small subunit r-proteins. We report the identification of Nap1 and Tsr4 as direct binding partners of Rps6 and Rps2, respectively. Both factors promote the solubility of their r-protein clients *in vitro*. While Tsr4 is specific for Rps2, Nap1 has several interaction partners including Rps6 and two other r-proteins. Tsr4 binds co-translationally to the essential, eukaryote-specific N-terminal extension of Rps2, whereas Nap1 interacts with a large, mostly eukaryote-specific binding surface of Rps6. Mutation of the essential Tsr4 and deletion of the non-essential Nap1 both enhance the 40S synthesis defects of the corresponding r-protein mutants. Our findings highlight that the acquisition of eukaryote-specific domains in r-proteins was accompanied by the co-evolution of proteins specialized to protect these domains and emphasize the critical role of r-protein chaperones for the synthesis of eukaryotic ribosomes.

## INTRODUCTION

Ribosomes are essential macromolecular machines responsible for protein synthesis in each cell. Considering that the entire population of ribosomes (∼200 000 in yeast) has to be duplicated each time a cell divides, ribosome synthesis is a central task in all growing cells (1). Eukaryotic ribosomes are composed of a large 60S and a small 40S subunit, which are assembled from their constituents, the ribosomal RNAs (rRNAs) and ribosomal proteins (r-proteins) in a highly conserved and complex maturation pathway orchestrated by several hundred different assembly factors (AFs; reviewed in (2–6)). At the beginning of this pathway stands, the synthesis of precursor rRNA (pre-rRNA) in the nucleolus, as well as the synthesis of r-proteins in the cytoplasm. Most r-proteins already associate, together with early-acting AFs, with the pre-rRNA in the nucleolus. There, the first ribosomal precursor particle, termed SSU processome or 90S particle, is formed and further converted into the successors in the pathway, the pre-40S and pre-60S particles.

The spatial separation of the sites of r-protein and pre-rRNA synthesis necessitates that most r-proteins are transported from the cytoplasm to the nucleolus. Consequently, a growing yeast cell has to import ∼150 000 r-proteins into the nucleus each minute (1), thus imposing an immense burden on the nuclear import machinery. On top of that, most r-proteins are highly basic, contain unstructured extensions, and are hence prone to aggregation as long as they are in their free form and not yet incorporated into ribosomes.

Several mechanisms have been reported to counteract aggregation of newly synthesized r-proteins. In addition to the general cellular chaperone network (7,8), also proteins of the nuclear import machinery (termed importins) protect r-proteins from aggregation by binding to their positively charged nuclear import sequences (NLSs) (9). Furthermore, we and others have realized in recent years that, in addition to these general mechanisms, at least some r-proteins resort to their own specific chaperones, which protect them from aggregation on their path to their rRNA assembly sites (10–21). These dedicated r-protein chaperones are a diverse group of proteins exploiting different structures and binding modes to protect their r-protein partners (14). Until now, eight such dedicated chaperones have been described.

Rrb1, Sqt1, Acl4 and Bcp1 are dedicated chaperones of the large ribosomal subunit r-proteins Rpl3, Rpl10, Rpl4 and Rpl23 (uL3, uL16, uL4 and uL14 according to the recently proposed new nomenclature of r-proteins (22)), respectively (10,13,16–20). Syo1 acts as a chaperone that binds two 60S r-proteins at the same time, Rpl5 and Rpl11 (uL18 and uL5) (21). Yar1 and Tsr2 are dedicated chaperones of the 40S subunit r-proteins Rps3 (uS3) and Rps26 (eS26), respectively (11,15). Moreover, there is evidence that Fap7 may act as a chaperone for the 40S r-protein Rps14 (uS11) (23,24). Additionally, it has been reported that Fap7 can act in an alternative pathway as a chaperone of a pre-assembled Rps14/Rps26 complex (12).

Together, the current knowledge suggests that dedicated r-protein chaperones are important contributors to the efficient assembly of newly synthesized r-proteins into nascent ribosomes. In contrast to the nine r-proteins for which a dedicated chaperone has already been described, no dedicated chaperones are known for the remaining ∼70 r-proteins. Given the immediate importance of r-protein chaperones for efficient ribosome assembly, we consider it likely that many r-proteins do actually have chaperones that have not been discovered yet.

To identify such novel dedicated r-protein chaperones, we performed a systematic screen in which we analyzed affinity-purifications of all 40S r-proteins by label-free semi-quantitative mass spectrometry. We then selected two r-proteins, Rps2 (uS5) and Rps6 (eS6), for further analyses and identified Tsr4 and Nap1 as interaction partners that were enriched in the respective r-protein purification relative to all other r-protein purifications. We reveal that both Tsr4 and Nap1 directly interact with their r-protein clients and increase their solubility *in vitro*. Moreover, we introduce Tsr4 as a dedicated chaperone that binds co-translationally to the eukaryote-specific N-terminal extension of Rps2 and is essential for ribosome assembly. Nap1 employs an r-protein specific binding mode to interact with Rps6 but also with two large subunit r-proteins, Rpl39 and Rpl42. Its deletion enhances the 40S synthesis defect occurring upon *RPS6* copy number reduction. In summary, our findings highlight that r-protein chaperones, despite their variability with respect to the specificity, the binding modes, and the binding stages, are important factors ensuring the efficient synthesis of new ribosomes.

## MATERIALS AND METHODS

### Yeast strains and genetic methods

The *Saccharomyces cerevisiae* strains used in this study are W303 derivatives generated by deletion and tagging at the genomic locus using established methods (25,26) and are listed in Supplementary Table S1. For the screen to find novel dedicated r-protein chaperones, each r-protein of the small subunit was C-terminally TAP-tagged. For duplicated r-proteins carrying two copies (A and B), only one copy (usually the one known to be higher expressed) was tagged. As the C-terminal TAP-tag fusion of the single copy r-protein Rps15 was not viable, we used a diploid strain in which only one allele of *RPS15* was tagged. Yeast and *Escherichia coli* plasmids were constructed using standard recombinant DNA techniques and are listed in Supplementary Table S2. All DNA fragments amplified by PCR were verified by sequencing.

### Plasmid shuffle assays

Shuffle strains were constructed by knocking out an essential gene in a diploid yeast strain, transformation with a *URA3* plasmid containing the respective wild-type gene, and sporulation to generate haploids harboring the gene knockout and the complementing *URA3* plasmid.

These shuffle strains were transformed with *LEU2* or *TRP1* plasmids carrying different alleles of the gene of interest. Subsequently, the ability of the transformants to grow after loss of the *URA3* plasmid on 5-FOA (Thermo Scientific) containing plates was evaluated. Strains that were viable on 5-FOA plates were subsequently analyzed for their growth phenotypes on plates lacking leucine or tryptophan (SDC –leu or –trp).

The *TSR4 RPS2* shuffle strain contained knockouts of both essential genes and a *URA3-RPS2* plasmid, which was sufficient to complement both knockouts. This strain was transformed with combinations of *LEU2* and *TRP1* plasmids carrying different alleles of *TSR4* and *RPS2*. After loss of the *URA3*-*RPS2* plasmid on 5-FOA containing plates, the growth of the strains was evaluated on SDC –leu –trp plates.

### Random PCR mutagenesis

Mutagenesis of the *TSR4* as well as *RPS2* open reading frames (ORFs) was performed using PCR reactions containing 25 μM MnCl_2_ for *RPS2* and 50 μM MnCl_2_ for *TSR4*. The resulting mutagenized ORF of *TSR4* was cloned into a *LEU2* plasmid whereas the mutagenized ORF of *RPS2* was cloned into a *TRP1* plasmid, both between the non-mutagenized promotor and terminator region of the respective genes. The resulting mutagenized libraries were transformed into the corresponding shuffle strains, containing chromosomal deletions of the respective gene complemented by a *URA3* plasmid carrying the wild-type gene. After loss of the *URA3*-wild-type plasmid by counter-selection on 5-FOA containing plates, mutants were screened for temperature-sensitive phenotypes as well as for general growth defects. Mutants with interesting phenotypes (*rps2-1, rps2-2, tsr4-1* and *tsr4-2*) were subjected to plasmid isolation and the contained mutations were identified by sequencing. The *rps2-1* allele encodes the exchange D106>G. The *rps2-2* allele harbors exchanges of four conserved amino acids (K49>E, L58>M, K64>E and Q89>L). The *tsr4-1* allele encodes two amino acid exchanges (P60>A and E398>G). The *tsr4-2* allele encodes only one amino acid exchange (R314>S).

### Tandem-affinity purification (TAP)

Yeast cells expressing C-terminal TAP-tag fusions of the r-proteins Asc1, Rps0a, Rps1b, Rps2, Rps3, Rps4b, Rps6a, Rps7b, Rps8a, Rps9a, Rps10a, Rps12, Rps13, Rps15, Rps17a, Rps18b, Rps19a, Rps20, Rps24b, Rps25a, Rps26b, Rps27a, Rps30a and Rps31, as well as the W303 control strain (untagged), were grown at 30°C in 4 l yeast extract peptone dextrose medium (YPD) to an optical density (OD_600_) of 2. Cells expressing Rps11b-, Rps14b-, Rps21a- and Rps29a-TAP were grown in 8 l YPD as above.

TAP purifications were performed in a buffer containing 50 mM Tris–HCl (pH 7.5), 100 mM NaCl, 1.5 mM MgCl_2_, 0.1% NP-40 and 1 mM dithiothreitol (DTT). Prior to use, 1× Protease Inhibitor Mix FY (Serva) was added freshly to the buffer. Cells were lysed by mechanical disruption using glass beads and the lysate was incubated with 300 μl IgG Sepharose™ 6 Fast Flow (GE Healthcare) at 4°C for 60 min. After incubation, beads were transferred into Mobicol columns (MoBiTec) and washed with buffer. Elution from IgG Sepharose™ beads was performed via TEV protease under rotation at room temperature for 90 min. After addition of 2 mM CaCl_2_, TEV eluates were incubated with 300 μl Calmodulin Sepharose™ 4B (GE Healthcare) at 4°C for 60 min. After washing with 2 ml buffer containing 2 mM CaCl_2_, followed by a second washing step with 5 ml 2 mM CaCl_2_ alone, proteins were eluted from Calmodulin Sepharose™ with 600 μl 0.8% ammonium hydroxide solution (Sigma) under rotation at room temperature for 20 min. One third of the eluates were dried via SpeedVac^®^ (Savant) and dissolved in SDS sample buffer. The protein samples were separated on NuPAGE™ 4–12% Bis–Tris gels (Invitrogen) followed by staining with NOVEX^®^ Colloidal Blue Staining Kit (Invitrogen). For LC–MS/MS analysis, the quantity of the eluates was adjusted according to the intensity of the Coomassie-stained bands and adjusted samples were dried via SpeedVac^®^.

Yeast cells expressing C-terminally TAP-tagged Tsr4 were grown at 30°C in 4 l YPD to an optical density (OD_600_) of 2. TAP purification was performed as mentioned above until elution with TEV protease.

### Nap1-TAP Rps6a-Flag split purification

Yeast cells expressing Nap1-TAP Rps6a-Flag were grown at 30°C in 8 l YPD to an OD_600_ of 2. TAP purification was performed as described above until elution with TEV protease. 50% of the TEV eluate was incubated with Calmodulin Sepharose™ 4B (GE Healthcare), whereas the other 50% of the TEV eluate was incubated with Anti-FLAG^®^ M2 Affinity Gel (Flag-beads, Sigma) at 4°C for 60 min. After washing the Calmodulin beads with 2 ml buffer containing 2 mM CaCl_2_, followed by a second washing step with 5 ml 2 mM CaCl_2_ alone, proteins were eluted with 600 μl 0.8% ammonium hydroxide solution (Sigma) under rotation at room temperature for 20 min (Nap1-TAP). After binding of the TEV eluate to Anti-FLAG^®^ M2 Affinity Gel the supernatant was collected and bound to Calmodulin Sepharose™ 4B at 4°C for 60 min after adding 2 mM CaCl_2_, whereas the Anti-FLAG^®^ M2 Affinity Gel was washed with lysis buffer, and proteins were eluted with 600 μl 0.8% ammonium hydroxide solution (Sigma) under rotation at room temperature for 20 min (Rps6a-enriched). After washing the Calmodulin beads with 2 ml buffer containing 2 mM CaCl_2_, followed by a second washing step with 5 ml 2 mM CaCl_2_ alone, proteins were eluted with 600 μl 0.8% ammonium hydroxide solution (Sigma) under rotation at room temperature for 20 min (Rps6a depleted). Protein samples were dried by SpeedVac^®^, dissolved in SDS sample buffer, and separated on NuPAGE™ 4–12% Bis–Tris gels (Invitrogen) followed by NOVEX^®^ Colloidal Blue Staining Kit (Invitrogen) and western blotting. For LC–MS/MS analysis, the eluates were dried by SpeedVac^®^.

### Western blotting

Western blot analysis was performed using the following antibodies: α-CBP antibody (1:5000; Merck–Millipore, cat. no. 07-482), horseradish peroxidase conjugated α-HA antibody (1:5000; Roche, cat. no. 12013819001), α-c-Myc antibody (1:1000; Sigma, cat. no. M5546), α-GAPDH antibody (1:40 000; Cell Signaling Technology, cat. no. 2118S), horseradish peroxidase conjugated α-Flag antibody (1:15 000; Sigma, cat. no. A8592), α-H2B antibody (1:5000; Abcam, cat. no. ab1790), α-Rps3 antibody (1:50 000; provided by Matthias Seedorf), α-Rps26/Tsr2 antibody (1:2000; provided by Vikram Panse), α-Rpl35 antibody (1:35 000; provided by Matthias Seedorf), α-Enp1 antibody (1:4000; provided by Katrin Karbstein), α-Rps2/Rpl30 antibody (1:2000; provided by Jonathan Warner), secondary α-rabbit horseradish peroxidase-conjugated antibody (1:15 000; Sigma, cat. no. A0545), secondary α-mouse horseradish peroxidase-conjugated antibody (1:10 000; Sigma, cat. no. NA931).

Protein signals were visualized using the Clarity™ Western ECL Substrate Kit (Bio-Rad) and captured by ChemiDoc™ Touch Imaging System (Bio-Rad).

### LC–MS/MS analysis

Eluates from the TAP purification were dissolved in 25% 2,2,2-trifluoroethanol (TFE) in 50 mM Tris–HCl (pH 8.5), reduced with 10 mM Tris(2-carboxyethyl)phosphine (TCEP) and alkylated with 40 mM chloroacetamide by shaking at 550 rpm at 95°C for 10 min. After dilution to <10% TFE with 50 mM ammonium bicarbonate, proteins were digested by adding 1 μg of Promega modified trypsin and shaking overnight at 550 rpm at 37°C. The resulting peptide solution was acidified by adding 5% formic acid to a final concentration of 0.1% and analyzed by nano-HPLC (Dionex Ultimate 3000, equipped with a C18 (5 μm, 100 Å, 5 × 0.3 mm) enrichment column and an Acclaim PepMap RSLC C18 nanocolumn (2 μm, 100 Å, 500 × 0.075 mm) (all Thermo Fisher Scientific)), coupled to tandem mass spectrometry.

Samples from the 40S r-protein TAP screen were concentrated on the enrichment column for 6 min at a flow rate of 5 μl/min with 0.1% heptafluorobutyric acid as isocratic solvent. Separation was carried out on the nanocolumn at a flow rate of 300 nl/min at 60°C using the following gradient, where solvent A is 0.1% formic acid in water and solvent B is acetonitrile containing 0.1% formic acid: 0–6 min: 4% B; 6–94 min: 4–25% B; 94 -99 min: 25–95% B, 99–109 min: 95% B; 109–124 min: 4% B. The maXis II ETD mass spectrometer (Bruker) was used as detector and operated with the captive source in positive mode with the following settings: mass range 200–2000 *m/z*, 2 Hz, capillary 1300 V, dry gas flow 3 l/min with 150°C, nanoBooster 0.2 bar, precursor acquisition control top17 (CID). The LC–MS/MS data were pre-processed by the Data analysis software (Bruker), using the Sum Peak algorithm.

Samples from the Nap1-TAP Rps6a-Flag experiment were concentrated on the enrichment column for 6 min using 0.1% formic acid as isocratic solvent at 5 μl/min flow rate. The column was then switched in the nanoflow circuit, and the sample was loaded on the nanocolumn, at a flow rate of 250 nl/min at 60°C and separated using the following gradient: solvent A: water, 0.1% formic acid; solvent B: acetonitrile, 0.1% formic acid; 0–6 min: 4% B; 6–264 min: 4–25% B; 264–274 min: 25–95% B, 274–289 min: 95% B; 289–304 min: 4% B. The sample was ionized in the nanospray source equipped with stainless steel emitters (Thermo Fisher Scientific) and analyzed in a Thermo Orbitrap Velos Pro mass spectrometer in positive ion mode by alternating full scan MS (*m/z* 300–2000, 60 000 resolution) in the ICR cell and MS/MS by CID of the 10 most intense peaks in the ion trap with dynamic exclusion enabled.

### Processing of LC–MS/MS data

LC–MS/MS data were analyzed by MaxQuant (versions: 1.5.3.30 for the 40S r-protein TAP screen and 1.6.0.16 for the Nap1-TAP Rps6a-FLAG experiment) by searching the public Swissprot database with taxonomy *S. cerevisiae* and common contaminants (downloaded on 24 August 2016 for the 40S r-protein TAP screen and 08 January 2018 for the Nap1-TAP Rps6a-FLAG experiment). Carbamidomethylation on Cys was entered as fixed modification, oxidation on methionine as variable modification. Detailed search criteria were used as follows: trypsin, max. missed cleavage sites: 2; search mode: MS/MS ion search with decoy database search included; precursor mass tolerance ±0.006 Da for Bruker data and ±4.5 ppm for Thermo data; product mass tolerance ±40 ppm for Bruker data and ±0.5 Da for Thermo data; acceptance parameters for identification: 1% PSM FDR; 1% protein FDR. In addition a label free quantitation of each protein calculated from the areas under the curve of precursor ion intensity chromatograms was performed using MaxQuant (27) requiring a minimum of two ratio counts of quantified razor and unique peptides. Relative protein intensities (% of total intensity per sample) are reported in Supplementary Tables S3 and S4.

### Graphical representation of LC–MS/MS data

#### 40S r-protein TAP screen

For a rough categorization, each detected protein was manually assigned to one of the following groups: 40S r-protein, 60S r-protein, ribosome AF, translation factor, other. The determined intensity values were normalized so that the sum of intensities was 100% in each purification. All 0-values were replaced by 0.0001 to allow conversion into logarithmic values used for the graphical representation of the data. For a rough overview of all data, the relative intensities were transformed to base-10 logarithmic values and the proteins were first sorted according to their group assignment followed by the mean signal of all purifications (with the higher signals on top). Subsequently, a heat map was generated using the Genesis software (28).

For detailed analysis of individual purifications, the relative intensities in the respective purification were compared to the mean intensities calculated from all purifications. The intensities obtained from the Rps3- and Rps14-TAP purifications were omitted from the mean calculations (except for the representations of the Rps3-TAP and the Rps14-TAP data) due to strong variation of these samples from the other samples. The scatter plots were generated in Statgraphics 18 using logarithmic scaling.

#### Nap1-TAP Rps6a-Flag purification

For a rough categorization, each detected protein was manually assigned to one of the following groups: 40S r-protein, 60S r-protein, ribosome AF, translation factor, cell division, histone, transcription, other. The determined intensity values were normalized so that the sum of all intensities measured in each purification, excluding the Nap1 intensity, was 100%. All 0-values were replaced by 0.0001 to allow for logarithmic display of the data.

The values from the Nap1-TAP, Rps6a-Flag enriched purification were plotted against the Nap1-TAP, Rps6a-Flag depleted purification in Statgraphics 18 using logarithmic scaling.

### Mass spectrometry for identification of proteins contained in gel bands

The protein bands were excised from the gel and then reduced, alkylated, and digested with Promega modified trypsin according to the method described in (29). Peptide extracts were dissolved in 0.1% formic acid, 5% acetonitrile and separated by nano-HPLC (Dionex Ultimate 3000) equipped with a C18 (5 μm, 100 Å, 5 × 0.3 mm) enrichment column and an Acclaim PepMap RSLC C18 nanocolumn (2 μm, 100 Å, 500 × 0.075 mm) (all Thermo Fisher Scientific, Vienna, Austria). Samples were concentrated on the enrichment column for 6 min at a flow rate of 5 μl/min employing 0.1% formic acid as isocratic solvent. Separation was carried out on the nanocolumn at a flow rate of 250 nl/min at 60°C using the following gradient, where solvent A is 0.1% formic acid in water and solvent B is acetonitrile containing 0.1% formic acid: 0–6 min: 4% B; 6–94 min: 4–25% B; 94–99 min: 25–95% B, 99–109 min: 95% B; 109-109.1 min: 95-4% B; 109.1–124 min: 4% B. The sample was ionized in the nanospray source equipped with stainless steel emitters (ES528, Thermo Fisher Scientific, Vienna, Austria) and analyzed in a Orbitrap Velos Pro mass spectrometer (Thermo Fisher Scientific, Waltham, MA, USA) operated in positive ion mode, applying alternating full scan MS (*m/z* 400–2000) in the ion cyclotron and MS/MS by CID of the 20 most intense peaks with dynamic exclusion enabled. The LC–MS/MS data were analyzed by searching a database containing all SwissProt *S. cerevisiae* sequences and all common contaminants (downloaded 08 January 2018, 8038 sequences) with Mascot 2.4.1 (MatrixScience, London, UK). Detailed search criteria: enzyme: semi-Trypsin, maximum missed cleavage sites: 2, Cys modification: carbamidomethylation, possible multiple oxidized methionine; precursor mass tolerance 10 ppm, product mass tolerance ±0.7 Da., 5% false discovery rate. Data were filtered according to stringent peptide acceptance criteria, including Mascot Ion Score of at least 20 and a position rank 1 in Mascot search.

### Yeast two-hybrid

Protein-protein interactions were analyzed by yeast two-hybrid (Y2H) assays using the reporter strain PJ69-4A, which allows detection of weak (*HIS3* reporter) and strong interactions (*ADE2* reporter). Two plasmids were co-transformed into the strain, whereby one plasmid was expressing the bait protein fused to the Gal4 DNA-binding domain (G4BD, BD, *TRP1* marker), followed by a c-Myc-tag, and the other the prey protein fused to the Gal4 transcription activation domain (G4AD, AD, *LEU2* marker), followed by an HA-tag. Colonies were spotted onto plates lacking leucine and tryptophan (SDC –leu –trp), lacking leucine, tryptophan, and histidine, (SDC –leu –trp –his, *HIS3* reporter) and lacking leucine, tryptophan, and adenine (SDC –leu –trp –ade, *ADE2* reporter), respectively. Plates were incubated at 30°C for 3 days.

For the competition experiment, PJ69-4A was first transformed with either a high-copy *URA3* plasmid expressing *RPS6* (pADH195-*RPS6A*) or an empty control plasmid (YEplac195); these two strains were then grown in liquid SDC medium lacking uracil (SDC –ura) and co-transformed with the bait and prey plasmids. To select transformants containing all three plasmids and to score the Y2H interactions, the above described media additionally lacked uracil.

To detect soluble expression of Gal4 DNA-binding or activation domain fusions, cells were grown in 20 ml SDC –leu –trp to an OD_600_ of ∼0.5. Cell pellets were suspended in ∼1.5-fold volume of lysis buffer (100 mM NaCl, 50 mM Tris–HCl, pH 8.0, 0.5 mM PMSF, 1× FY protease inhibitor) and lysed by glass bead lysis and an aliquote of the lysate was taken. Glass beads were subsequently removed by centrifugation for 2 min at 380 × g. The resulting supernatant was centrifuged for 10 min at 18 000 × g to remove insoluble material. After addition of SDS sample buffer, lysate, supernatant, and pellet fractions were analyzed by western blotting using α-c-Myc (G4BD fusion proteins) and α-HA (G4AD fusion proteins) antibodies.

### Protein co-expression in *E. coli* and *in vitro* binding assays

For protein expression, the *RPS2-*His6 (MCS1), Flag-*RPS6A* (MCS2) and *NAP1* (MCS1) genes, as well as the combination of Flag-*RPS6A* and *NAP1*, were cloned into the pETDuet-1 vector (Novagen), respectively. *TSR4*-Flag (MCS2) was cloned into the pCOLADuet-1 vector (Novagen) and plasmids were transformed into an *E. coli* BL21 (DE3) Rosetta strain (Novagen). Cells were grown in 2 l lysogeny broth (LB) medium at 30°C to an OD_600_ of 0.3–0.4 before protein expression was induced with 0.3 mM isopropyl-β-d-thiogalactoside (IPTG, Thermo Scientific). Cells expressing Rps6, Nap1, and the combination of both were further incubated at 30°C for 2 h. Cells expressing Rps2, Tsr4 and the combination of both were shifted to 25°C upon IPTG addition and incubated for 20 h. Cell pellets were resuspended in lysis buffer containing 50 mM Tris–HCl (pH 7.5) and 150 mM NaCl. Prior to use, 60 mM imidazole, 1× Protease Inhibitor Mix HP (Serva), 0.5 mM phenylmethanesulfonyl fluoride (PMSF, Sigma), 1 mM dithiothreitol (DTT) and 1 mg/ml lysozyme (Roth) was added and cells were lysed via sonication. After centrifugation cell lysates were incubated with 400 μl Ni-NTA agarose (Qiagen) at 4°C for 60 min. Beads were washed three times with 5 ml washing buffer (lysis buffer containing 1 mM DTT and 60 mM imidazole) before being transferred into Mobicol columns (MoBiTec). After a second washing step with 5 ml washing buffer, bound proteins were eluted with 400 μl buffer containing 300 mM imidazole under rotation at 4°C for 20 min. Eluates were incubated with 300 μl Anti-FLAG^®^ M2 Affinity Gel (Sigma) under rotation at 4°C for 60 min. Beads were washed three times with 1 ml lysis buffer containing 1 mM DTT and transferred into Mobicol columns followed by a washing step with 5 ml buffer. Bound material was eluted with 300 μl buffer containing 100 μg/ml FLAG^®^ peptide (Sigma). Samples were analyzed via NuPAGE™ 4–12% Bis-Tris gels (Invitrogen), and stained with NOVEX^®^ Colloidal Blue Staining Kit (Invitrogen).

### *In vitro* protein solubility assay in *E. coli*

Cells were grown in 50 ml LB medium at 30°C to an OD_600_ of 0.3–0.4 before protein expression was induced with 0.3 mM IPTG as described above. 60 OD_600_-units were harvested and resuspended in lysis buffer containing 50 mM Tris–HCl (pH 7.5), 150 mM NaCl, 1× Protease Inhibitor Mix HP (Serva), 0.5 mM PMSF (Sigma), 1 mM DTT and 1 mg/ml lysozyme (Roth). Cells were disrupted by sonication and samples were taken (input). After centrifugation (15 000 rpm, 4°C for 30 min), supernatants and pellets were separated and dissolved in SDS sample buffer. 0.1% of the protein samples were separated on NuPAGE™ 4–12% Bis-Tris gels (Invitrogen) followed by staining with NOVEX^®^ Colloidal Blue Staining Kit (Invitrogen).

### Sucrose gradient analysis

Cells for the experiment shown in Figure 4E were grown in SDC medium at 37°C to an OD_600_ of ∼0.5. Cells for the experiment shown in Figure 6F were grown in 70 ml SDC medium lacking leucine and tryptophan (SDC –leu –trp) at 25°C to an OD_600_ of ∼0.25 and shifted to 37°C for 2 h. 100 μg/ml cycloheximide was added to 50 ml culture and cells were incubated on ice for 5 min. Cells were pelleted and resuspended in lysis buffer containing 10 mM Tris–HCl (pH 7.5), 100 mM NaCl, 30 mM MgCl_2_ and 100 μg/ml cycloheximide. After cell lysis using glass beads, 4–5 *A*_260_ units of the cell extracts were loaded onto 5–45% sucrose gradients and centrifuged at 38 000 rpm in a SW41Ti rotor at 4°C for 2 h 45 min using a Beckman Optima™ LE-80K ultracentrifuge. Gradients were analyzed using a UA-6 system (Teledyne Isco) with continuous monitoring at *A*_254_ nm.

### Co-translational binding assay

Co-translational association of C-terminally TAP-tagged chaperones (Yar1-TAP, Fap7-TAP, Tsr2-TAP, Tsr4-TAP and Nap1-TAP) with nascent r-proteins was assessed by IgG-Sepharose pull-down and qRT-PCR as previously described (30) with the following modifications: (i) the volumes of the yeast cultures (200 ml) and the IgG-Sepharose beads (50 μl) were decreased by half, and cycloheximide was added to a final concentration of 0.1 mg/ml to the cultures and buffers; (ii) after DNase treatment (DNA-*free*™ Kit DNase Treatment & Removal Kit, Thermo Fisher Scientific), RNA samples were diluted 40 times and 3 μl of these dilutions were used to perform the qRT-PCR using the KAPA SYBR^®^ FAST One-Step Universal kit (Merck) according to the manufacturer's instructions; (iii) the raw data were analyzed using the program LinRegPCR (31), and the starting concentration values (N0), expressed in arbitrary fluorescence units, were used for further calculation. For each mRNA amplicon, the average N0 values of the technical triplicates of each TEV eluate were first divided by the average N0 values of the technical triplicates of the corresponding total extract. To correct for the background levels, the mean values of the mRNA abundance ratios (TEV/total), excluding the mRNA corresponding to the respective r-protein binding partner, were first calculated for each respective chaperone purification (mean purification background value) and each mRNA amplicon (mean amplicon background value). The individual mRNA abundance ratios (TEV/total) were then divided by both their corresponding mean purification and amplicon background values. Normalized mRNA abundance ratios were obtained by setting the mean of all corrected background values to 1.

The following oligonucleotide pairs were used for the specific amplification of DNA fragments, corresponding to the *RPS2, RPS3, RPS6, RPS14*, and *RPS26* mRNAs, from the input cDNAs: RPS2-forward 5′-AGGGATGGGTTCCAGTTACC-3′ and RPS2-reverse 5′-TGGCAAAGAGTGCAAGAAGA-3′ (amplicon size 89 base pairs (bp)), RPS3-forward 5′-GCTGCTTACGGTGTCGTCAGAT-3′ and RPS3-reverse 5′-AGCCTTAGCTCTGGCAGCTCTT-3′ (amplicon size 96 bp), RPS6-forward 5′-CAAGGCTCCAAAGATCCAAA-3′ and RPS6-reverse 5′-TGAGCGTTTCTGACCTTCAA-3′ (amplicon size 87 bp), RPS14-forward 5′-TCCATACGCTGCTATGTTGG-3′ and RPS14-reverse 5′-TCTTAACGTGAACGGCAGTG-3′ (amplicon size 80 bp), and RPS26-forward 5′-TCCAAAGGATAAGGCTATCAAGA-3′ and RPS26-reverse 5′-AGAAGCTTCGGACAAATCTCTG-3′ (amplicon size 82 bp).

### Fluorescence microscopy

Yeast cells in logarithmic growth phase were imaged by fluorescence microscopy using a Leica DM6 B microscope, equipped with a DFC 9000 GT camera, using the PLAN APO 100× objective and the LasX software.

A plasmid containing the *RPS2*-3xyEGFP fusion gene was transformed into a *NOP58*-RedStar2 Δ*tsr4* Δ*rps2* [*URA3*-*RPS2*] strain, and, as control, into a *NOP58*-RedStar2 Δ*rps2* [*URA3*-*RPS2*] strain. The *RPS2*-3xyEGFP fusion was not fully functional, as it was (in contrast to a *LEU2* plasmid containing *RPS2* without GFP fusion) not sufficient to suppress the Δ*tsr4* phenotype, preventing loss of the *URA3*-*RPS2* plasmid in a Δ*tsr4* or a Δ*tsr4*Δ*rps2* strain. Therefore, we investigated the localization of the Rps2-3xyEGFP fusion in the presence of the *URA3*-*RPS2* plasmid.

To estimate the proportion of cells with nuclear signal or nuclear exclusion, ∼30–45 microscopic images, including in total at least 1000 cells with a visible Nop58-RedStar2 signal (used to determine the position of the nucleolus), were analyzed per strain. All cells in which the GFP-signal in the nucleus was at least as strong as in the cytoplasm were counted as cells with nuclear signal, whereas cells with clearly less signal in the nucleus than in the cytoplasm were counted as cells with nuclear exclusion.

## RESULTS

### A screen to identify new dedicated chaperones of 40S r-proteins

**Figure 1.**
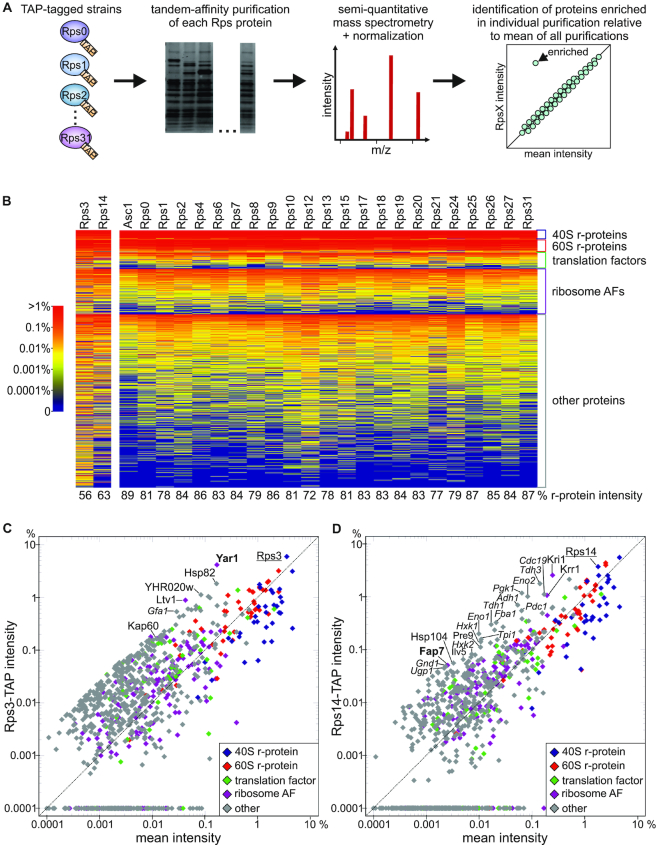
A screen to identify new dedicated chaperones of 40S r-proteins. (**A**) Overview of the screening strategy. (**B**) Heat map of proteins detected in the indicated 40S r-protein TAP purifications, sorted according to protein groups and mean intensities. The color bar on the left indicates the colors corresponding to the relative intensities. At the bottom, the calculated percentages of the sum of intensities of all r-proteins relative to the total measured intensities in the respective samples are indicated. (**C** and **D**) Relative intensities in the Rps3- (**C**) and Rps14- (**D**) TAP purification plotted against the mean intensity of all purifications. The respective bait protein (underlined) and proteins particularly enriched in the purification are labeled, with the respective known dedicated chaperones displayed in bold and contaminants implicated in carbohydrate metabolism indicated in italics.

To identify dedicated chaperones among the many interaction partners common to all r-proteins (i.e. other r-proteins, translation factors, etc.), we established a tandem-affinity purification (TAP) based screen (see Figure 1A). Since 75% of all r-proteins are encoded by two paralogous genes (A and B copy) in yeast (32), we constructed strains, by genomic tagging of either the single copy genes or one of the two paralogs (indicated in Supplementary Table S1), individually expressing each small subunit r-protein fused to a C-terminal TAP-tag and purified r-proteins together with their interaction partners by TAP purification. Eight r-proteins (Rps5 (uS7), Rps11 (uS17), Rps16 (uS9), Rps22 (uS8), Rps23 (uS12), Rps28 (eS28), Rps29 (uS14) and Rps30 (eS30)) were purified inefficiently, thus preventing further analyses (Supplementary Figure S1A and data not shown). Eluates from the remaining 25 purifications were subjected to label-free semi-quantitative mass spectrometry. Most r-protein purifications yielded a pattern characteristic of mature ribosomes, with most co-purifying proteins migrating in the low-molecular-weight range, suggesting that the r-proteins were efficiently incorporated into ribosomes despite the C-terminal TAP-tag (Supplementary Figure S1A). This was also reflected by the measured intensities for r-proteins, which made up between 72% (Rps12 (eS12)) and 89% (Asc1 (RACK1)) of the total measured intensities in the samples (Figure 1B, Supplementary Table S3). Only for the less well incorporated Rps3- and Rps14-TAP, r-proteins made up only 56% and 63% of the total signal in the samples, respectively.

To assess if their known chaperones were co-enriched with Rps3 and Rps14, we plotted the intensities of each protein detected in the Rps3-TAP and Rps14-TAP purifications against the mean intensity calculated from the signals of the same protein in all purifications (Figure 1C and D). In this representation, all proteins detected at similar intensities as in all other purifications are expected to lie along a diagonal with the slope of 1, while proteins that are enriched in the respective purifications should be positioned clearly above this diagonal. The diagrams for Rps3-TAP and Rps14-TAP showed a relatively broad spread of dots, probably due to the reduced co-purification of r-proteins, along with an increased enrichment of contaminations. Nevertheless, the Rps3-TAP purification resulted as expected in a strong co-enrichment of its dedicated chaperone Yar1 (Figure 1C and Supplementary Figure S1B). Moreover, the AF Ltv1, which is also a direct partner of Rps3 (33,34), was clearly enriched in this purification. We previously found that Rps3 mainly uses the ‘classical’ import pathway employing yeast importin-α Kap60/Srp1 and importin-β Kap95 for its nuclear import (35). Indeed, we observed that also Kap60 was enriched in the Rps3-TAP purification relative to other r-protein purifications, supporting our previous findings.

Moreover, we also found the putative Rps14 chaperone Fap7 enriched in the Rps14-TAP purification (Figure 1D and Supplementary Figure S1B). Additionally, also the AF Kri1 and, to a lesser extent, its direct partner Krr1 (36) were enriched in this purification. Notably, Rps14 and Krr1 interact with each other in 90S particles (37–39). Moreover, Krr1 forms a complex together with Rps14 and Fap7 *in vitro* and it was suggested that Fap7 might promote the assembly of Rps14 and Krr1 into 90S particles or, alternatively, that Krr1 may recruit the Rps14/Fap7 complex to 90S particles (40). It will be interesting to address in the future whether also Krr1’s partner Kri1 participates in this maturation step.

The third 40S r-protein known to have a dedicated chaperone is Rps26 (15). In our screen, we detected the Rps26 chaperone Tsr2 neither in the Rps26-TAP purification nor in any of the other purifications. Moreover, also Fap7, which was reported to act alternatively to Tsr2 and to chaperone a pre-formed Rps14/Rps26 complex (12), was not detected in the Rps26-TAP purification (Supplementary Figure S1B). We speculate that the interactions of Rps26 with Tsr2 and with Fap7 might be too short-lived to be detected in our approach.

To sum up, our screen is suitable to uncover direct partners of individual r-proteins, including dedicated chaperones, although some transient interactions may escape detection by this method.

### Tsr4 and Nap1 are direct binding partners of the r-proteins Rps2 and Rps6

Next, we focused our analyses on 40S r-proteins for which no dedicated chaperone has been reported so far. We selected three r-proteins, Rps6, Rps2, and Rps15, that bind pre-40S particles at different positions and maturation stages, and plotted the intensities of each protein recovered in these purifications against the mean of all purifications (excluding the outliers Rps3- and Rps14-TAP).

**Figure 2.**
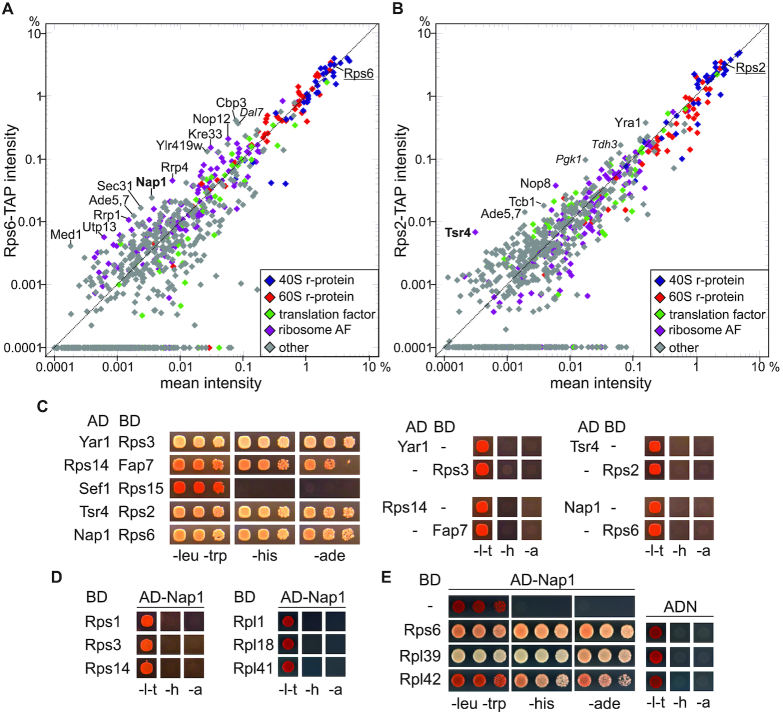
Tsr4 and Nap1 are direct binding partners of Rps2 and Rps6. (**A** and **B**) Relative intensities in the Rps6- (**A**) and Rps2- (**B**) TAP purification plotted against the mean intensity of all purifications. The respective bait protein (underlined) and proteins particularly enriched in the purification are labeled, with contaminants implicated in carbohydrate metabolism indicated in italics. The putative novel r-protein chaperones Nap1 and Tsr4 are displayed in bold. (**C**–**E**) Yeast two-hybrid (Y2H) assays of the indicated combinations of Gal4 activation domain (AD) and Gal4 DNA-binding domain (BD) fusions. Growth on SDC –leucine –tryptophan –histidine plates (labeled –his or –h) indicates a weak interaction; growth on SDC –leucine –tryptophan –adenine plates (labeled –ade or –a), as well as white colored colonies on SDC –leucine –tryptophan (labeled –leu–trp or –l–t) plates are indications of a strong Y2H interaction. All orientations of activation and binding domain fusions were tested and only one representative combination is shown. (**C**) Direct interaction between Tsr4 and Rps2, as well as Nap1 and Rps6. The proteins Yar1, Fap7, and Nap1 were N-terminally fused to the indicated Gal4-domain, whereas Rps3, Rps14, Sef1, Rps15, Tsr4, Rps2, and Rps6 were fused C-terminally. In the right panels, negative controls either expressing only the respective activation or binding domain fusion together with empty plasmid (AD or BD on their own) as depicted are shown. (**D**) Nap1 shows no interaction with Rps1, Rps3 Rps14, Rpl1, Rpl18 or Rpl41 in the Y2H assay. (**E**) Nap1 interacts not only with Rps6, but also with Rpl39 and Rpl42. Rpl39 was fused N-terminally and Rpl42 C-terminally to the BD. On the right panel, negative controls expressing the indicated BD fusions in combination with empty plasmid (AD only) are represented.

**Rps6** (eS6) is an early-binding 40S r-protein (41,42) associating with the early-folding 5′-domain of the 18S rRNA, which forms the ‘body’ of 40S particles (Supplementary Figure S2A). It has two paralogs, Rps6a and Rps6b, with identical amino-acid sequence. Among the factors co-enriched with Rps6 were a few known AFs, including Kre33, which directly interacts with Rps6 within 90S particles (Figure 2A) (37,38). Its co-purification may be an indication that Rps6 and Kre33 are incorporated into pre-ribosomes together or that Kre33 even facilitates Rps6 incorporation. Additionally, Nap1, although present in most r-protein purifications, was clearly enriched in a few of them, including Rps6-TAP (Figure 2A and Supplementary Figure S1B). Nap1 was described to have a dual function as histone chaperone and regulator of cell division at the septin ring (43–49). Moreover, the *Arabidopsis thaliana* Rps6 (*at*Rps6) orthologue was reported to interact with *Arabidopsis* Nap1, whereby *at*Rps6 was suggested to have an extra-ribosomal, histone-related function together with Nap1 (50). Our study indicates, however, that Nap1 may also have a ribosome-related function as chaperone for Rps6 and/or other r-proteins (see below).

**Rps2** (uS5) is positioned at the junction between the body of 40S particles and the later folding 3′-major domain of the 18S rRNA, which forms the ‘head’ of 40S particles (Supplementary Figure S2A). It can be detected by western blotting in 90S particles purified via Krr1-TAP (34), suggesting it associates at the stage of 90S particles. Its binding to pre-ribosomes is important for the efficient release of AFs Rrp12 and Slx9 from 40S precursors, as well as for efficient binding of the AF Rio2 (51). In the Rps2-TAP purification, one of the most enriched proteins was Tsr4 (Figure 2B). Tsr4 is a ∼46 kDa, acidic protein (p*I* ∼4.5), which is conserved among eukaryotes (Supplementary Figure S5A). Tsr4 was discovered in a systematic study to be a new ribosome AF whose depletion leads to 40S subunit maturation defects (52). However, its exact function in the ribosome biogenesis pathway was up to now unknown. Moreover, recent studies suggested that the human Tsr4 orthologue PDCD2L, its human paralog PDCD2, and the *Drosophila* Tsr4 orthologue Zfrp8 interact with Rps2 (53,54). These data further support our idea that Tsr4 might be a novel chaperone of Rps2.

**Rps15** (uS19) is a late-associating r-protein (41,42), binding to the head domain of pre-40S particles (Supplementary Figure S2A). A few proteins were enriched in the Rps15-TAP purification, with Sef1, a largely uncharacterized protein, being the most prominent one (Supplementary Figure S2B).

To test whether proteins enriched in individual r-protein purifications are capable of directly interacting with the respective bait r-proteins, we subjected promising r-protein/putative chaperone pairs (*i.e*. Rps6/Nap1, Rps2/Tsr4 and Rps15/Sef1) to a yeast two-hybrid (Y2H) assay (Figure 2C). Indeed, this analysis indicated a direct interaction between Rps2 and Tsr4, as well as between Rps6 and Nap1. No Y2H interaction was detected between Rps15 and Sef1, suggesting that Sef1 either interacts indirectly with Rps15 via another factor, or that it was a false positive in our screen, or a false negative in the Y2H assay.

For the rest of our study, we focused on the further characterization of the two proteins that scored positive in the Y2H, i.e. Nap1 and Tsr4, which we considered to be promising candidates for novel r-protein chaperones of Rps6 and Rps2, respectively. In contrast to Tsr4, which was only detected in the Rps2-TAP purification, Nap1 was also found, albeit to a lesser extent, in most other r-protein purifications (Supplementary Figure S1B). To address whether Nap1 interacts exclusively with Rps6, we assessed by Y2H potential interactions of Nap1 with other r-proteins, which were either in this study (Supplementary Figure S1B) or previously (44,55–57) suggested to be associated with Nap1. None of the other tested 40S r-proteins (i.e. Rps1 (eS1), Rps3, and Rps14) interacted with Nap1 in this assay. Moreover, the large subunit r-proteins Rpl1 (uL1), Rpl18 (eL18), and Rpl41 (eL41), which were found in large-scale studies to interact with Nap1 (44,55,56), showed no Y2H interaction with Nap1, suggesting they do not interact directly (Figure 2D). In contrast however, two 60S subunit r-proteins, Rpl39 (eL39) and Rpl42 (eL42), which were also identified as Nap1 partners in large-scale studies (55,56), did interact with Nap1 in the Y2H assay (Figure 2E). We conclude that Nap1 directly interacts with Rps6 and at least two other r-proteins.

### Nap1 employs an r-protein specific binding mode and protects Rps6 from aggregation *in vitro*

**Figure 3.**
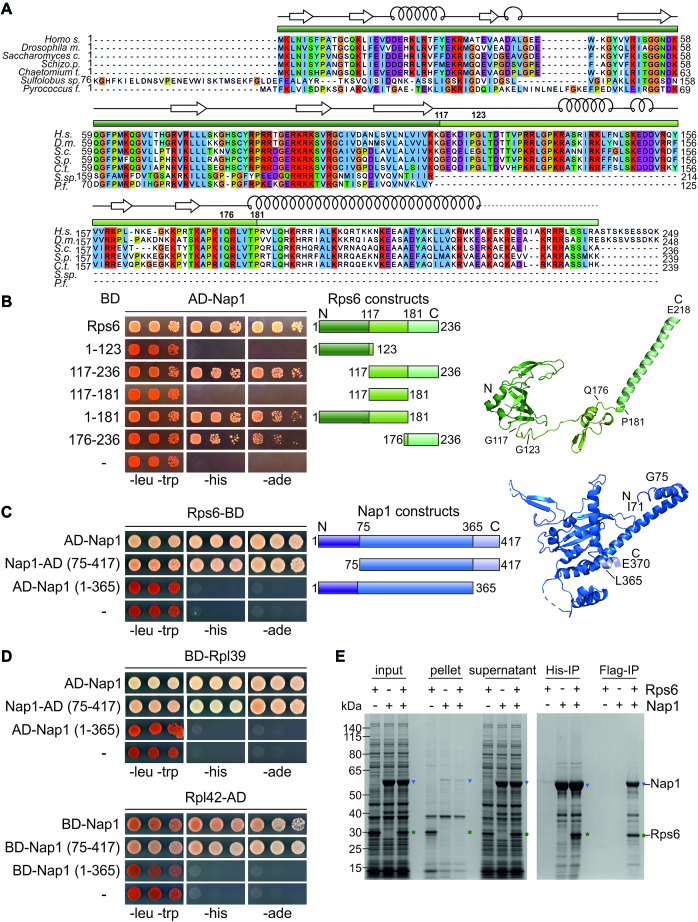
Nap1 employs an r-protein specific binding mode and protects Rps6 from aggregation *in vitro*. (**A**) Sequence alignment of Rps6 reveals a eukaryote specific C-terminal part. Sequences were aligned with Clustal Omega and viewed in Jalview. Domains and secondary structure elements are indicated as in the protein structure (B). *Sulfolobus species* contains an N-terminal extension, which is missing in other species, therefore the *Sulfolobus* 75 N-terminal amino acids are not shown in the alignment. (**B**–**D**) Y2H assays of Nap1 with Rps6, Rpl39, or Rpl42. For negative controls, see Supplementary Figure S3B and S3C. (**B**) Rps6 reveals a complex binding surface for the interaction with Nap1. Rps6 and Rps6 fragments were C-terminally fused to the Gal4 DNA-binding domain (BD), whereas full-length Nap1 was N-terminally fused to the Gal4 activation domain (AD). The different constructs of Rps6 are schematically depicted on the right in the corresponding color schemes and marked in the structure (extracted from PDB 4V88 (71)). (**C** and **D**) The unstructured C-terminus of Nap1 is essential for binding to Rps6 (**C**), Rpl39 and Rpl42 (**D**). The different truncations of Nap1 are schematically depicted on the right (C) and indicated in the structure (PDB 2AYU (68)), as well as in the alignment of Nap1 (Supplementary Figure S3A). A comparison of the interactions dependent on N- or C-terminal tagging of Nap1 truncations is shown in Supplementary Figure S4A. (**E**) Coomassie-stained gel of the solubility assay and *in vitro* binding assay of Nap1 and Rps6. Proteins were (co-)expressed in *E. coli*, cells were lysed (input), centrifuged at 40 000 x g and the supernatant fraction (soluble proteins) was separated from the pellet (insoluble material). Rps6 (marked in green pentagons) expressed on its own aggregated and was mostly found in the pellet fraction, whereas in combination with Nap1 (indicated in blue triangles), the r-protein was soluble and stayed in the supernatant. The two-step affinity purification via His6-Nap1 followed by Flag-Rps6 resulted in the co-purification of both proteins indicating that Nap1 and Rps6 form a complex *in vitro*.

To better understand which part of Rps6 is bound by Nap1, we mapped the interacting domains via Y2H analysis. Rps6 is present in eukaryotes and archaea, but absent in bacteria. It contains a conserved N-terminal beta-barrel domain, which is connected by a eukaryote-specific loop region with a long, eukaryote-specific C-terminal alpha-helix (Figure 3A and B). While neither the N-terminal domain nor the loop region of Rps6 were able to interact with Nap1 by themselves, the C-terminal domain (residues 176–236) interacted with Nap1, although the interaction was weaker than with full-length Rps6 (Figure 3B). Almost full interaction was reached when the Rps6 C-terminal domain was combined with the loop region (residues 117–236). Notably, also combination of the N-terminal domain of Rps6 with the loop (residues 1–181) resulted in an interaction with Nap1. We conclude that Rps6 has a complex binding surface containing several Nap1-binding regions, with the eukaryote-specific C-terminal Rps6 alpha-helix likely comprising the main interaction surface. Notably, archaea, which lack the Rps6 loop and C-terminal domain, also lack an orthologue of Nap1.

Next, we investigated Nap1 truncations for interaction with Rps6 (Figure 3C, Supplementary Figures S3C and S4A). Nap1 has unstructured extensions both in its N- and C-terminal parts (Supplementary Figure S3A). While removal of the 74 N-terminal residues of Nap1 (residues 75–417) still allowed almost full interaction with Rps6, the interaction with Rps6 was completely abolished when the 52 C-terminal amino acids were removed (residues 1–365). Notably, this C-terminal extension (residues 366–417) is neither required for the binding of Nap1 to histones (48,58,59) nor to the importin Kap114 (47). To assess if this different binding mode is common to r-proteins, we also tested the same Nap1 truncations for interaction with Rpl39 and Rpl42 (Figure 3D, Supplementary Figures S3C and S4A). Indeed, also for these interactions, the unstructured N-terminal region of Nap1 was dispensable, while its unstructured C-terminal extension was required. We conclude that Nap1 exhibits a common binding mode when interacting with r-proteins, for which the acidic C-terminal extension, which is dispensable for interactions with other partners, is critical. To test if the r-proteins indeed compete for the same binding site within Nap1, we assessed the strength of the Y2H interactions between Nap1 and Rpl39 or Rpl42 in cells expressing either endogenous or increased levels of Rps6. This analysis revealed that overexpression of Rps6 from a multicopy plasmid clearly reduced, but did not abolish, the interaction between Nap1 and Rpl39 or Rpl42 (Supplementary Figure S4B), further corroborating that all three r-proteins bind to a common surface on Nap1.

Next, we examined the *in vitro* interactions between Nap1 and Rps6. When we expressed Rps6 alone, large amounts of the protein were insoluble (Figure 3E). Importantly, co-expression of Nap1 shifted the r-protein almost completely to the soluble fraction, suggesting that Nap1 has the capability to increase the solubility of its partner Rps6. Moreover, Nap1 and Rps6 were co-purified in a two-step affinity purification, revealing that they also form a complex *in vitro* (Figure 3E). Together, our Y2H and *in vitro* interaction analyses indicate that Nap1 directly interacts with Rps6 and thereby increases the solubility of the r-protein.

### Nap1 functions together with Rps6 *in viv*o

**Figure 4.**
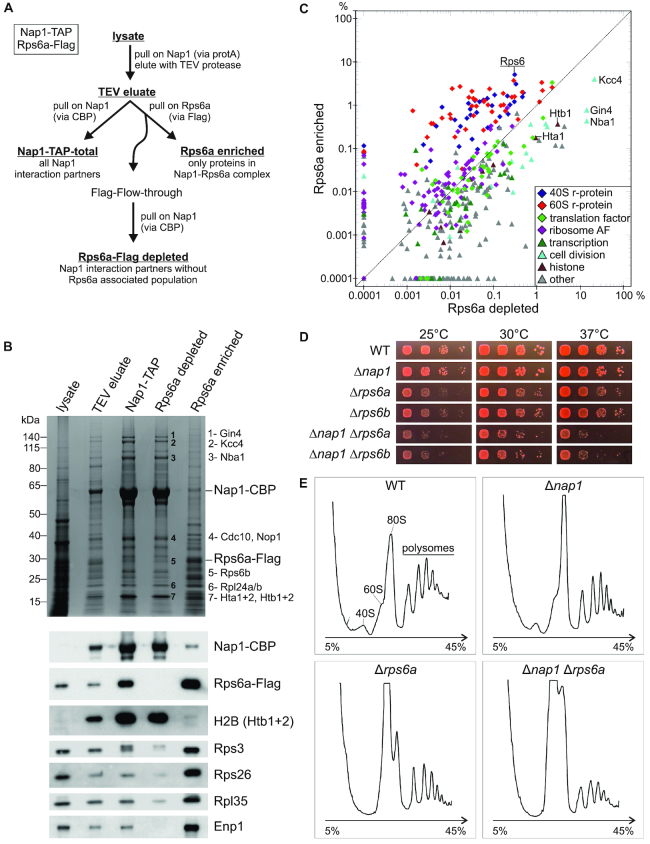
Nap1 functions together with Rps6 *in vivo*. (**A**) Schematic representation of the Nap1-TAP Rps6a-Flag split purification. (**B**) Split-tag purifications were performed as indicated in (A) and all samples written in bold and underlined names were analyzed via SDS-PAGE, followed by Coomassie staining or Western blotting. Nap1-CBP was detected via α-CBP antibody, Rps6a-Flag with α-Flag antibody; all other proteins were detected with antibodies specific to the respective protein as indicated. Protein bands analyzed by mass spectrometry are numbered and listed on the right. (**C**) Relative intensities in the Rps6a-Flag enriched sample plotted against the relative intensities in the Rps6a-Flag depleted sample. Rps6a and the most prominent interaction partners of Nap1 (see also (B)) are indicated. (**D**) *NAP1* and *RPS6* are genetically linked. Cells were spotted on SDC plates and incubated at the indicated temperatures for 2 days. (**E**) Nap1 has a function in ribosome biogenesis together with Rps6. Yeast cells of the wild-type strain (WT) and the knockout strains Δ*nap1*, Δ*rps6a*, and Δ*nap1* Δ*rps6a* were grown in SDC at 37°C, lysed after inhibition of translation with cycloheximide and polysome profiles were recorded. Peaks corresponding to the 40S and 60S subunit, 80S and polysomes are indicated in the profile of the wild-type strain.

To characterize the complexes in which Nap1 and Rps6 occur together, we generated a strain expressing TAP-tagged Nap1 and a Flag-tag fusion of one of the two Rps6 paralogs, Rps6a (Figure 4A). First, we performed purification of Nap1-TAP via IgG-Sepharose, followed by TEV elution, and incubated the resulting eluate (comprising all Nap1-containing complexes) with anti-Flag beads to purify only the sub-population containing both Nap1-CBP and Rps6a-Flag. In addition, we used the supernatant from this incubation (Nap1-containing complexes depleted of complexes containing Rps6a-Flag; note that Rps6b was still present in these complexes) and purified them in a second step via Calmodulin beads. In parallel, we also performed a standard TAP purification of Nap1, yielding all partners of Nap1. Finally, we analyzed the resulting eluates by label-free semi-quantitative mass spectrometry, as well as by SDS-PAGE followed by Coomassie-staining and Western blotting (Figure 4B and C). Indeed, increased Rps6a-Flag amounts were present in the Rps6a-Flag enriched sample, whereas Rps6a-Flag was absent from the Rps6a-Flag depleted sample (Figure 4B).

The overall pattern of the Rps6a-Flag depleted purification resembled the standard Nap1-TAP purification, showing a strongly over-stoichiometric Nap1-containing band on the Coomassie-stained gel, accounting for more than 50% of the total intensity in the mass spectrometric analysis of the sample (Supplementary Table S4). The most prominent co-purifying proteins in the total Nap1-TAP and the Rps6a-Flag depleted purification were the functionally redundant kinases Gin4 and Kcc4, both known to interact with Nap1 in the context of cell division regulation, and Nba1, another less well characterized partner of Nap1 (43,44). Last but not least, there was a strong band in the range of ∼15 kDa containing histones H2B, H2AZ and H2A (proteins Htb2, Htz1, and Hta1) in both purifications (Figure 4B and C). Additionally, we detected the AF Enp1, a marker protein for 90S and pre-40S particles, in the Nap1-TAP purification, but its association was severely reduced in the Rps6a-Flag depleted sample, as obvious from the Western blot analysis (Figure 4B).

In the Rps6a-Flag enriched purification, the protein composition was significantly changed compared to the other two purifications. While r-proteins and ribosome AFs, including Enp1, were enriched, histones and proteins implicated in cell division were under-represented in the Rps6a-Flag enriched pool (Figure 4B and C). We speculate that Nap1 and Rps6a co-exist together in a (pre-)ribosomal complex. The observation that 40S and 60S r-proteins were more co-enriched than ribosome AFs is somewhat surprising and may be either due to the presence of contaminating mature ribosomes in the TEV eluate, which are subsequently further co-purified with Rps6a-Flag, or alternatively, may be an indication that Nap1 is also present in mature ribosomes. Furthermore, Western blotting indicated that Rps6a-Flag was clearly more enriched in the Nap1-TAP purification than other r-proteins (compare relative levels of Rps6, Rps3, Rps26 and Rpl35 in the lysate and the Nap1-TAP eluate), suggesting that Rps6 and Nap1 also exist as a free complex together.

### *nap1* mutants enhance the ribosome assembly defect of *rps6a* mutants

Having established that Nap1 is a direct binding partner of Rps6, we wanted to determine if the combination of *rps6* and *nap1* mutations leads to an enhancement of growth defects, which would be a further indication for a functional connection between these two proteins. *NAP1* deletion strains did not show any growth defects; however, the growth defects of both the *RPS6A* and *RPS6B* knockout strains were enhanced at 37°C when in addition *NAP1* was deleted (Figure 4D). We conclude that *NAP1* and *RPS6* show a genetic interaction.

To address whether these enhanced growth phenotypes go along with a ribosomal defect, we recorded polysome profiles of the Δ*nap1* strain, the Δ*rps6*a strain, and the Δ*nap1* Δ*rps6a* double knockout mutant (Figure 4E). Notably, the Δ*nap1* strain showed an increased 80S peak and slightly reduced polysome levels, hence ribosomal subunits are capable of joining into 80S ribosomes, but these engage in translation less efficiently. This may either be the consequence of the synthesis of not fully functional ribosomal subunits in the absence of Nap1, or might be due to a problem in translation initiation itself. The Δ*rps6*a knockout strain showed a drastic reduction of free 40S subunits, accompanied by a significant increase of the 60S peak, a phenotype typical for defects causing the reduced production of 40S subunits, consequently resulting in an excess of 60S subunits. When in addition to *RPS6A* also *NAP1* was deleted, a further increase of the 60S peak could be observed, suggesting an even stronger 40S maturation defect. Moreover, the 80S peak was increased compared to the Δ*rps6a* mutant alone, indicating the additional presence of a translation initiation defect (that was also observed in the Δ*nap1* single mutant). Last but not least, polysome levels of the double mutant were significantly reduced compared to the single mutants. Together, our results reveal that Nap1 has a so far unanticipated function together with Rps6 in ribosome biogenesis.

### Tsr4 binds co-translationally to the N-terminal extension of Rps2

**Figure 5.**
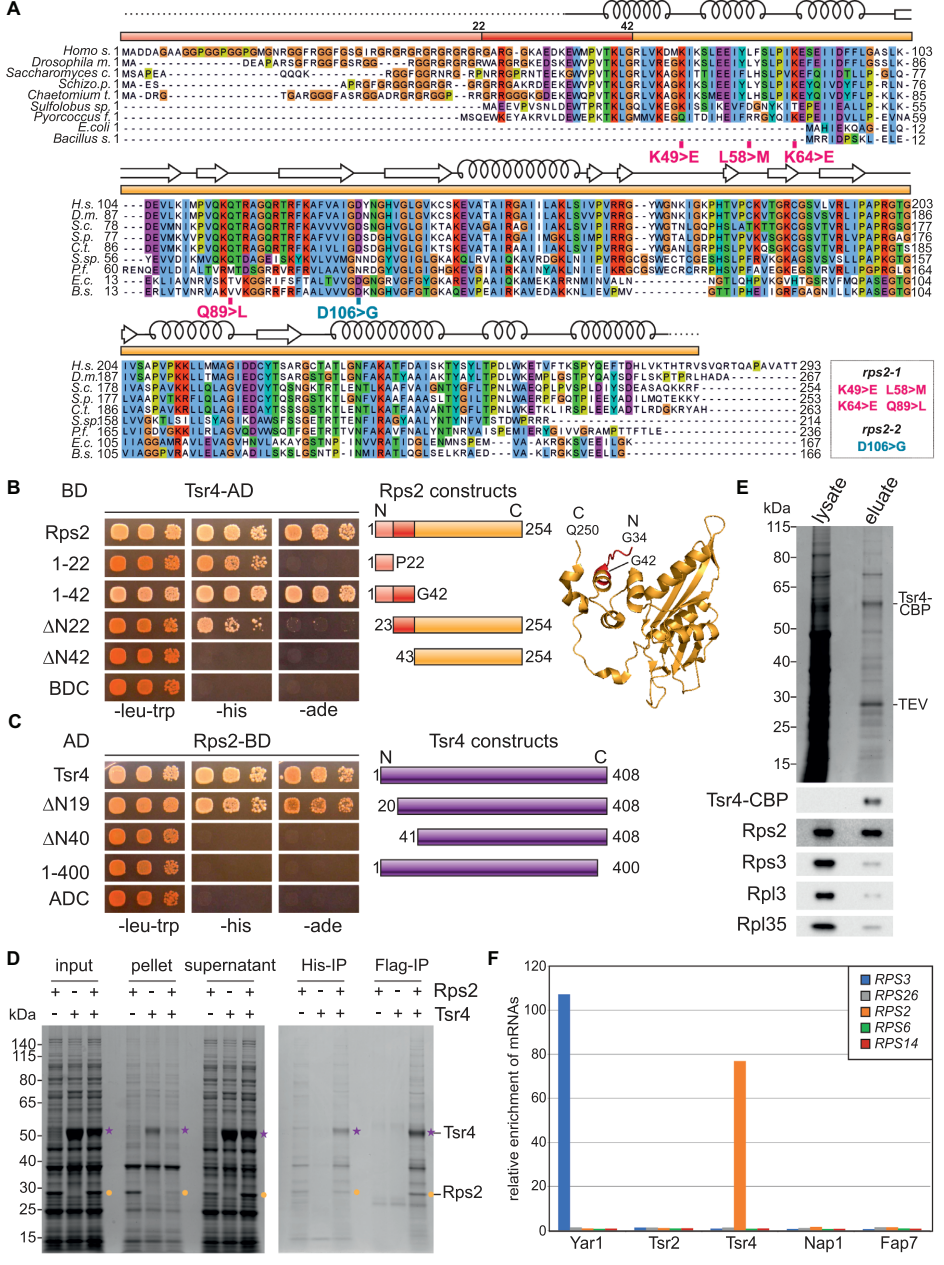
Tsr4 binds co-translationally to the N-terminus of Rps2. (**A**) Sequence alignment of Rps2 reveals a eukaryote-specific N-terminus. Sequences were aligned with Clustal Omega and viewed in Jalview; domains are sketched as colored in the structure (B). Truncations of Rps2 used in this study are indicated above the sequence accordingly, mutations introduced into Rps2 are marked in the sequence and listed on the bottom right. (**B**) Tsr4 binds to the eukaryote-specific N-terminal extension of Rps2. In this Y2H experiment, Rps2 and Rps2 truncations were C-terminally fused to the Gal4 DNA-binding domain (BD), Tsr4 was C-terminally fused to the Gal4 activation domain (AD). Constructs of Rps2 are schematically depicted on the right and fragment borders are indicated in the structure (extracted from PDB 4V88 (71)). For negative controls, see also Supplementary Figure S5B. (**C**) A large part of the dedicated chaperone Tsr4 is needed for the Y2H interaction with Rps2. Tsr4 and truncations of Tsr4 were C-terminally fused to AD, and Rps2 was C-terminally fused to BD. The different truncations of Tsr4 are schematically depicted on the right. Negative controls are shown in Supplementary Figure S5C. (**D**) Coomassie-stained gel of the solubility assay and *in vitro* binding assay of Tsr4 and Rps2. Proteins were (co-)expressed in *E. coli*, cells were lysed (input), centrifuged at 40 000 x g and the supernatant fraction (soluble proteins) was separated from the pellet (insoluble material). Rps2 (marked in yellow dots) expressed on its own aggregated and was found mostly in the pellet fraction, whereas in combination with Tsr4 (indicated in purple stars), the r-protein was soluble and stayed in the supernatant. The two-step affinity purification via Rps2-His6 followed by Tsr4-Flag resulted in the co-purification of both proteins indicating that Tsr4 and Rps2 form a complex *in vitro*. (**E**) Tsr4 and Rps2 form a complex *in vivo*. Affinity purification of Tsr4-TAP via IgG-Sepharose, followed by Western blotting, reveals co-purification of Rps2. Tsr4-CBP was detected via α-CBP antibody; all other proteins were detected with antibodies specific to the respective protein as indicated. (**F**) Tsr4 associates co-translationally with Rps2. TAP-tagged Yar1, Tsr2, Tsr4, Nap1 and Fap7 were affinity purified via IgG-sepharose after translation inhibition by cycloheximide. After RNA extraction, the indicated mRNAs were detected via qRT-PCR.

In order to characterize Tsr4, our dedicated chaperone candidate for Rps2, we first mapped the Tsr4 interaction region on Rps2 by Y2H. Rps2 protein homologs exist in eukaryotes, archaea and bacteria; eukaryotes however contain a short N-terminal extension that is missing in prokaryotes (Figure 5A). Notably, these N-terminal 42 amino acids of Rps2 were sufficient for its Y2H interaction with Tsr4, while a Rps2 variant lacking these residues was not capable of interacting with Tsr4 (Figure 5B and Supplementary Figure S5B). Moreover, both the first 22 amino acids of Rps2 and the corresponding N-terminal deletion variant (ΔN22, residues 23–254) exhibited a weak interaction, hence elements before amino acid 23 and between amino acids 23 and 42 are required for full interaction with Tsr4.

Next, we mapped the Rps2 interacting region of Tsr4 and found that while deletion of the 19 N-terminal amino acids of Tsr4 (ΔN19, residues 20–408) still allowed almost full interaction with Rps2, removal of the 40 N-terminal residues (ΔN40, residues 41–408) abolished the interaction (Figure 5C and Supplementary Figure S5C). Moreover, the interaction was completely lost when the eight conserved C-terminal amino acids of Tsr4 (residues 1–400; Supplementary Figure S5A) were missing. Notably, all constructs were expressed and soluble, although they were detected at slightly (Tsr4 N-terminally truncated fragments) or significantly (Tsr4 C-terminally truncated fragment) reduced levels compared to full-length Tsr4 (Supplementary Figure S5E). As however not even a weak Y2H interaction with Rps2 was detected with the Tsr4 ΔN40 and the Tsr4 1–400 variants, despite the presence of soluble protein, we conclude that both the N- and the C-terminal part of Tsr4 are required for the interaction with Rps2.

To further confirm the direct interaction between Tsr4 and Rps2, we also performed an *in vitro* interaction assay (Figure 5D). As described above for Nap1 and Rps6, we observed that co-expression of Tsr4 increased the solubility of Rps2 expressed in *E. coli*. Moreover, both proteins co-purified in a two-step purification, indicating they form a complex *in vitro*. In order to examine the *in vivo* interactions of Tsr4, we affinity-purified TAP-tagged Tsr4 from yeast and analyzed the resulting eluate (Figure 5E). Tsr4 was the only prominent band visible in the Coomassie-stained gel; however, Western blotting indicated an enrichment of Rps2 in the purification compared to other r-proteins, further supporting that Tsr4 and Rps2 form a complex in yeast cells.

The interaction of Tsr4 with the very N-terminus of Rps2 is reminiscent of several other r-protein chaperones, i.e. Yar1, Syo1, Rrb1 and Sqt1 (14). All these chaperones are known to associate with their r-protein partners already co-translationally (13,30). To assess whether this is also the case for Tsr4, we performed affinity-purification of TAP-tagged Tsr4 after translation inhibition by cycloheximide, retaining translating ribosomes on the mRNAs, and detected associated mRNAs by quantitative reverse transcription PCR (qRT-PCR) (Figure 5F). If Rps2 is already bound by Tsr4 co-translationally, not only the Rps2 protein, but also the *RPS2* mRNA is expected to co-purify with Tsr4-TAP in this approach. In addition to Tsr4, we also tested Nap1 and Fap7 for potential co-translational binding of Rps6 and Rps14, respectively. After TEV elution of the bait proteins together with associated proteins and RNAs, RNA was extracted and the levels of all mRNAs of interest (i.e. *RPS2, RPS6, RPS14, RPS3* and *RPS26*) were analyzed in all samples by qRT-PCR. Strikingly, purification of Tsr4-TAP significantly enriched the *RPS2* mRNA to a similar extent as Yar1-TAP enriched the *RPS3* mRNA (Figure 5F). We conclude that Tsr4 already binds co-translationally to the very N-terminal region of Rps2. In contrast, no co-translational binding of Nap1 or Fap7 to their clients could be detected; hence they might, like the escortin Tsr2 (15), associate with their r-protein partners post-translationally.

### Tsr4 is required for correct functioning of Rps2

**Figure 6.**
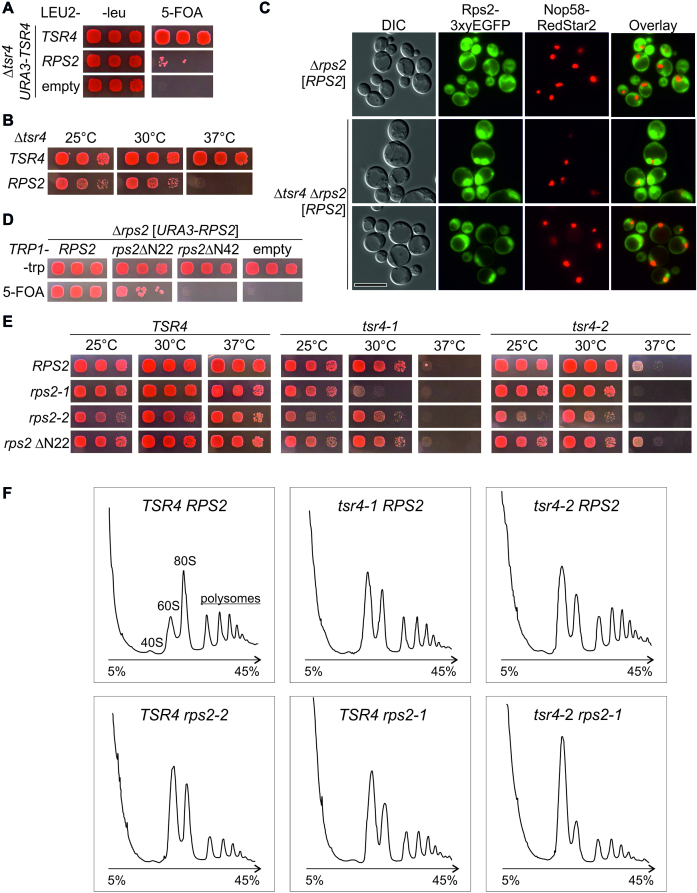
Tsr4 functions together with Rps2 in 40S subunit biogenesis. (**A** and **B**) Overexpression of *RPS2* overcomes the lethality of the *TSR4* knockout strain. (**A**) The capability of Δ*tsr4* strains, carrying the indicated plasmids, to lose the essential *URA3-TSR4* plasmid was determined by growth on 5-FOA containing plates, incubated at 30°C for 3 days. (**B**) *TSR4* knockout cells complemented by *TSR4* and *RPS2*, respectively, were spotted on SDC –leucine (–leu) plates and incubated at the indicated temperatures for 3 days. (**C**) In the absence of Tsr4, Rps2 accumulates in the nucleus. Fluorescence microscopy of Δ*rps2* and Δ*rps2* Δ*tsr4* strains, containing both a *URA3-RPS2* and a *LEU2-RPS2-*3xyEGFP plasmid, and a chromosomal C-terminal RedStar2-fusion of the nucleolar marker Nop58. The scale bar in the lower left panel represents 10 μm. In wild-type cells, C-terminally fused Rps2-3xyEGFP was located in the cytoplasm, whereas the nucleus was excluded. In contrast, in the absence of Tsr4, Rps2-3xyEGFP was detected also in the nucleus. The two different Δ*tsr4* panels reflect the variations in the extent of nuclear accumulation in different cells in the sample. Each panel was processed individually to make the observed phenotypes apparent. To allow evaluation of the differences in signal intensities, the same panels, but all identically processed, are shown in Supplementary Figure S6, panels A, C and D. (**D**) The N-terminal 42 amino acids of Rps2, which interact with Tsr4, are essential. The *rps2* knockout strain carrying *RPS2* on a *URA3* plasmid was transformed with wild-type *RPS2* and N-terminal truncations of *RPS2* on a *TRP1* plasmid or empty vector. Growth after loss of the *URA3*-*RPS2* plasmid was evaluated on 5-FOA containing plates. (**E**) Genetic enhancement of *rps2* and *tsr4* mutations. Δ*rps2* Δ*tsr4* cells carrying a combination of *LEU2* plasmids with *TSR4* variants and *TRP1* plasmids with *RPS2* variants were spotted on SDC –leucine –tryptophan (–leu –trp) plates and incubated at the indicated temperatures for 3 days. For phenotypes of Δ*tsr4* mutants alone, see Supplementary Figure S5D. (**F**) Tsr4 is crucial for 40S biogenesis. Δ*rps2* Δ*tsr4* cells complemented by plasmids carrying the indicated *TSR4* and *RPS2* variants were grown in SDC –leu –trp at 30°C and shifted to 37°C for 2 h. Cells were lysed after translation inhibition with cycloheximide and polysome profiles were recorded. Peaks corresponding to the 40S and 60S subunit, 80S and polysomes are indicated in the profile of the wild-type strain.

In several examples, growth defects caused by mutation or deletion of a dedicated r-protein chaperone can be compensated by the increased production of the client r-protein (14). Tsr4 is essential in the W303 strain background, as revealed by the inability of the *TSR4* shuffle strain, transformed with an empty plasmid, to grow on 5-FOA containing plates. Importantly, the presence of extra copies of *RPS2* provided on a low-copy plasmid supported viability of the Δ*tsr4* strain (Figure 6A). However, in contrast to the strain complemented with wild-type *TSR4*, the Δ*tsr4* strain rescued by *RPS2* showed a growth defect at 25°C and 30°C and was inviable at 37°C (Figure 6B). We conclude that an increased *RPS2* dosage can partially compensate for the absence of *TSR4*.

Next, we constructed a Δ*tsr4* Δ*rps2* strain complemented by a *URA3*-*RPS2* plasmid and containing a chromosomally encoded Nop58-RedStar2 fusion and transformed it with a *LEU2-RPS2*-3xyEGFP plasmid in order to investigate the localization of Rps2 in the absence of Tsr4 (Figure 6C and Supplementary Figure S6). As expected, in *TSR4* wild-type cells Rps2-GFP was localized in the cytoplasm, with ∼72% of the cells displaying a nuclear exclusion (Figure 6C, upper panel). Many of the Δ*tsr4* cells showed morphologic deficits, i.e. enlarged cells, enlarged vacuoles, and cells with budding defects (Supplementary Figure S6, panels C–E), consistent with the relatively strong growth defect of Δ*tsr4* cells upon suppression by plasmid encoded *RPS2* (Figure 6B). Notably, a nuclear signal of Rps2-GFP was observed in ∼82% of the Δ*tsr4* cells, sometimes with a massive accumulation of Rps2-GFP signal in the nucleus (Figure 6C, lower two panels, and Supplementary Figure S6, panels C–E). We conclude, that Rps2-GFP can be imported into the nucleus in the absence of Tsr4 but is most likely not efficiently assembled into pre-ribosomes.

To characterize the phenotype of *tsr4* mutants in the absence of the suppressing *RPS2* plasmid, and to compare them with *rps2* mutants, we then aimed at generating conditional *tsr4* and *rps2* mutants. Considering that the first 42 N-terminal amino acids of Rps2 are both necessary and sufficient for interaction with Tsr4 (Figure 5B), we first generated an *rps2* variant lacking the sequence encoding these N-terminal 42 amino acids (*rps2* ΔN42). This mutant was inviable (Figure 6D). In contrast, a mutant lacking only 22 N-terminal amino acids (*rps2* ΔN22), which still showed a weak Y2H interaction with Tsr4 (Figure 5B), was viable, but exhibited a mild growth defect (Figure 6D and E). In addition, we also generated *rps2* and *tsr4* alleles by random PCR mutagenesis, which we termed *rps2-1, rps2-2, tsr4-1* and *tsr4-2* (Figure 5A, Supplementary Figure S5A and D). Dedicated chaperone mutants frequently enhance the defects caused by mutation of the client r-protein (11,13,18,30). Indeed, all combinations of *tsr4* and *rps2* point mutations led to enhanced growth defects when compared to the growth of the single mutants, especially at higher temperatures, further corroborating that *TSR4* and *RPS2* interact genetically (Figure 6E). In contrast, the combination of the *rps2* ΔN22 deletion with the two *tsr4* alleles resulted in no significant enhancement of growth defects.

To assess the impact of this genetic interaction on ribosome biogenesis, we next examined the *tsr4* and *rps2* mutants by polysome profile analysis (Figure 6F). Indeed, not only the two *rps2* but also the two *tsr4* point mutants showed strong 40S synthesis defects, as revealed by the increased levels of free 60S subunits, and a reduction in overall translation as indicated by the decrease in 80S/monosome and polysome levels. Notably, the combination of the two milder *rps2-1* and *tsr4-2* single mutants resulted in a strongly enhanced defect, with a completely missing 40S peak, a large increase in 60S levels, and a prominent reduction in 80S and polysome content. We conclude that functional Tsr4 is crucial for efficient assembly of Rps2 into 40S particles and consequently for ribosome biogenesis.

## DISCUSSION

With this study, we have introduced two novel chaperones of r-proteins, Tsr4 and Nap1. These two proteins highlight nicely how diverse r-protein chaperones can be with respect to the binding mode, specificity, stage of association with the r-protein, and essentiality for cell survival. With the successive addition of new members, one can now look for common themes and accordingly subclassify r-protein chaperones.

Tsr4 belongs to the group of dedicated chaperones that associate with their client r-protein already co-translationally. As most other dedicated chaperones in this group, Tsr4 binds to the very N-terminal region of its r-protein partner Rps2. Notably, this N-terminal region of Rps2 seems to be flexible and was not resolved in any crystal or cryo-EM structures of (pre-)40S subunits so far. The unstructured nature of this part of the protein, together with the high content in positively charged arginines (Figure 5A) may explain the special requirement of this region for a chaperone.

*TSR4* is an essential gene, suggesting that chaperoning of Rps2 is an essential cellular task. Moreover, deletion of Rps2’s N-terminal 42 amino acids is lethal, probably either because Tsr4 cannot be recruited or because this N-terminal region has an important function itself and therefore needs to be protected by Tsr4. The co-translational binding of Tsr4 to Rps2 indicates that Rps2 is already captured by Tsr4 in the cytoplasm. Large-scale studies suggest that Tsr4 has a cytoplasmic steady-state localization (60), like the Rps3 chaperone Yar1 (11). The fact that Rps2-GFP accumulates in the nucleus in the absence of Tsr4 (Figure 6C), however, suggests that Tsr4 has very likely a nuclear function in Rps2 ribosome incorporation, while it is apparently not required for nuclear import of Rps2. In line with our results, the human Tsr4 homolog PDCD2L is a nucleo-cytoplasmic shuttling protein (53).

The second r-protein interactor we identified, Nap1, has very different characteristics. Nap1 is well described as a histone chaperone for H2A and H2B (47,61,62). R-proteins and histones have many common features, including a high content in positive charges and the need to be transported from the cytoplasm into the nucleus. Therefore, the utilization of the same protein for chaperoning histones and r-proteins seems plausible. Moreover, Nap1 is also promiscuous among r-proteins and can interact, apart from Rps6, also with the large subunit r-proteins Rpl39 and Rpl42. We showed that for the interaction of Nap1 with Rps6, Rpl39 and Rpl42, the C-terminal 52 amino acids of Nap1 are absolutely essential (Figure 3C and D). Remarkably, this region is not essential for the interaction with histones, although the presence of this region increases the affinity of Nap1 for histones (48,59,63–66). Therefore, despite the common features of histones and r-proteins, Nap1 employs a different interaction mode when binding to these various classes of proteins. Considering the acidic nature of Nap1’s C-terminal tail, it is tempting to speculate that the tail is used to shield positive charges of r-proteins.

Nap1 does not seem to bind Rps6 co-translationally, which is not surprising considering it recognizes a relatively large binding surface, with the C-terminal alpha-helix probably comprising the main interaction surface. Hence, Rps6 is probably already fully folded when it encounters its chaperone. Nap1 is known to be a nucleo-cytoplasmic shuttling protein (47,67). In its function as a histone chaperone, Nap1 is imported, together with histones H2A and H2B, into the nucleus by the importin Kap114 (47). We did not detect Kap114 in our Rps6-TAP nor in the Nap1-TAP purification, while low amounts of Kap123 were detected in both purifications (Supplementary Tables S3 and S4). The detailed investigation of the nuclear import pathway of Rps6, and of the stage at which Nap1 associates with Rps6, remains an interesting question for future studies. Notably, Nap1 is known to form dimers, not only in its free form, but also in the complex with histones (58,66,68). Finding out whether Nap1 is also dimeric when bound to r-proteins, and if Nap1 can bind more than one r-protein at the same time, will be another interesting subject for future studies.

Notably, Nap1 not only co-purifies r-proteins, but also ribosome AFs and translation factors (Figure 4B and C). Moreover, deletion of *NAP1* causes a mild translation initiation defect, but also enhances the 40S synthesis defect of a Δ*rps6a* strain (Figure 4E). These data show that the function of Nap1 is important for ribosome biogenesis and possibly also for translation. The engagement of Nap1 in several different pathways may indicate that it coordinates ribosome assembly with other key cellular processes.

Both Tsr4 and Nap1 have in common that they preferentially bind to eukaryote-specific regions of their r-protein clients. Similar observations have also been made for Acl4 and Tsr2 (13,16,69,70). Moreover, also the N-terminal region of Rpl3, where Rrb1 binds (30), and most of the Sqt1- and Syo1-binding regions within the Rpl10 and Rpl5 N-termini (21,30), respectively, are specific to eukaryotes (71).

We speculate that the acquisition of additional domains/extensions during evolution may have come, at least in some cases, with the cost of unwanted features, such as an increased aggregation tendency. This problematic situation is likely further aggravated by the longer residence time of r-proteins in their free forms due to the introduction of nucleo-cytoplasmic transport as an additional step in the ribosome assembly path of r-proteins in eukaryotes. Together, these developments may have favored the co-evolution of chaperones that protect the additional domains of eukaryotic r-proteins.

## Supplementary Material

gkz317_Supplemental_Files
