# Supplementary material for: Tsr4 and Nap1, two novel members of the ribosomal protein chaperOME
Source: Nucleic Acids Res. 2019 May 7;47(13):6984–7002. doi: 10.1093/nar/gkz317 (PMC6648895; doi:10.1093/nar/gkz317)
Supplement: gkz317_Supplemental_Files [file gkz317_supplemental_files.zip › RoesslerRevised_Supplements.pdf]

Supplementary Tables

Supplementary Table 1. Yeast strains

name	genotype	source
W303**a**	*MAT***a** *ade2-1, his3-11,15, leu2-3,112, trp1-1, ura3-1, can1-100*	(1)
W303**a**/**α**	*MAT***a**/*MAT***α** *ade2-1/ade2-1, his3-11,15/his3-11,15, leu2-3,112/leu2-3,112, trp1-1/trp1-1, ura3-1/ura3-1, can1-100/can1-100*	(1)
Asc1-TAP	*MAT***a** *ASC1*-TAP::HIS3MX4	this study
Rps0a-TAP	*MAT***a** *RPS0A*-TAP:: HIS3MX4	this study
Rps1b-TAP	*MAT***a** *RPS1B*-TAP:: HIS3MX4	this study
Rps2-TAP	*MAT***a** *RPS2*-TAP:: HIS3MX4	this study
Rps3-TAP	*MAT***a** *RPS3*-TAP::natNT2	(2)
Rps4b-TAP	*MAT***a** *RPS4B*-TAP:: HIS3MX4	this study
Rps6a-TAP	*MAT***a** *RPS6A*-TAP:: HIS3MX4	this study
Rps7b-TAP	*MAT***a** *RPS7B*-TAP:: HIS3MX4	this study
Rps8a-TAP	*MAT***a** *RPS8A*-TAP:: HIS3MX4	this study
Rps9a-TAP	*MAT***a** *RPS9A*-TAP:: HIS3MX4	this study
Rps10a-TAP	*MAT***a** *RPS10A*-TAP:: HIS3MX4	this study
Rps11b-TAP	*MAT***a** *RPS11B*-TAP:: HIS3MX4	this study
Rps12-TAP	*MAT***a** *RPS12*-TAP:: HIS3MX4	this study
Rps13-TAP	*MAT***a** *RPS13*-TAP:: HIS3MX4	this study
Rps14b-TAP	*MAT***a** *RPS14B*-TAP:: HIS3MX4	this study
Rps15-TAP diploid	*MAT***a**/*MAT***α** *RPS15*-TAP:: HIS3MX4/*RPS15*	this study
Rps17a-TAP	*MAT***a** *RPS17A*-TAP:: HIS3MX4	this study
Rps18b-TAP	*MAT***a** *RPS18B*-TAP:: HIS3MX4	this study
Rps19a-TAP	*MAT***a** *RPS19A*-TAP:: HIS3MX4	this study
Rps20-TAP	*MAT***a** *RPS20*-TAP:: HIS3MX4	this study
Rps21a-TAP	*MAT***a** *RPS21A*-TAP:: HIS3MX4	this study
Rps24b-TAP	*MAT***a** *RPS24B*-TAP:: HIS3MX4	this study
Rps25a-TAP	*MAT***a** *RPS25A*-TAP:: HIS3MX4	this study
Rps26b-TAP	*MAT***a** *RPS26B*-TAP:: HIS3MX4	this study
Rps27a-TAP	*MAT***a** *RPS27A*-TAP:: HIS3MX4	this study
Rps29a-TAP	*MAT***a** *RPS29A*-TAP:: HIS3MX4	this study
Rps30a-TAP	*MAT***a** *RPS30A*-TAP::HIS3MX4	this study
Rps31-TAP	*MAT***a** *RPS31*-TAP::HIS3MX4	this study
PJ69-4A	*MAT***a** *trp1-901 leu2-3,112 ura3-52 his3-200 gal4Δ gal80Δ LYS2*::*GAL1-HIS3 GAL2-ADE2 met2*::*GAL7*-lacZ	(3)
Nap1-TAP Rps6a-Flag	*MAT***a** *NAP1*-TAP::HIS3MX4 *RPS6A*-FLAG::natNT2	this study
Δ*nap1*	*MAT***a** *nap1*::HIS3MX4	this study
Δ*rps6a*	*MAT***a** *rps6a*:kanMX4	this study
Δ*rps6b*	*MAT***a** *rps6b*:kanMX4	this study

Δ*nap1* Δ*rps6a*	*MAT***a** *nap1*::HIS3MX4 *rps6a*:kanMX4	this study
Δ*nap1* Δ*rps6b*	*MAT***a** *nap1*::HIS3MX4 *rps6b*:kanMX4	this study
Tsr4-TAP	*MAT***a** *TSR4*-TAP::HIS3MX4	this study
TSR4 shuffle	*MAT***α** *tsr4*::HIS3MX4 [pRS316-*TSR4*]	this study
RPS2 shuffle	*MAT***α** *rps2*::kanMX4 [pRS316-*RPS2*]	this study
Δ*tsr4* Δ*rps2* *pRS316-RPS2*	*MAT***α** *tsr4*::HIS3MX4 *rps2*::kanMX4 [pRS316-*RPS2*]	this study
NOP58-RedStar2 *RPS2* shuffle	*MAT***α** *NOP58*-RedStar2::natNT2 *rps2*::kanMX4 [pRS316-*RPS2*]	this study
NOP58-RedStar2 Δ*tsr4* Δ*rps2* *pRS316-RPS2*	*MAT***a** *NOP58*-RedStar2::natNT2 *tsr4*::HIS3MX4 *rps2*::kanMX4 [pRS316-*RPS2*]	this study
Yar1-TAP	*MAT***α** *YAR1*-TAP::HIS3MX4	(4)
Tsr2-TAP	*MAT***a** *TSR2*-TAP-T*CYC1*::natNT2	this study
Fap7-TAP	*MAT***a** *FAP7*-TAP-T*CYC1*::natNT2	this study
Nap1-TAP	*MAT***a** *NAP1*-TAP::HIS3MX4	this study

All yeast strains used in this study, except PJ69-4A, are derivatives of W303.

Supplementary Table 2. *S. cerevisiae* and *E. coli* plasmids.

**name**	**relevant information**	**source**
pG4ADHAN111-*YAR1*	CEN, *LEU2*, P*ADH1*, T*ADH1,* N-terminal G4AD-HA	this study
pG4BDC22-*RPS3*	CEN, *TRP1*, P*ADH1*, T*ADH1,* C-terminal G4BD-c-Myc	(5)
pG4ADC111-*RPS14*	CEN, *LEU2*, P*ADH1*, T*ADH1,* C-terminal G4AD-HA	this study
pG4BDN22-*FAP7*	CEN, *TRP1*, P*ADH1*, T*ADH1,* N-terminal G4BD-c-Myc	this study
pG4ADC111-*SEF1*	CEN, *LEU2*, P*ADH1*, T*ADH1,* C-terminal G4AD-HA	this study
pG4BDC22-*RPS15*	CEN, *TRP1*, P*ADH1*, T*ADH1,* C-terminal G4BD-c-Myc	this study
pG4ADC111-*TSR4*	CEN, *LEU2*, P*ADH1*, T*ADH1,* C-terminal G4AD-HA	this study
pG4BDC22-*RPS2*	CEN, *TRP1*, P*ADH1*, T*ADH1,* C-terminal G4BD-c-Myc	this study
pGAG4ADC111-*NAP1*	CEN, *LEU2*, P*ADH1*, T*ADH1,* C-terminal $(GA)_5$-G4AD-HA	this study
pG4ADHAN111-*NAP1*	CEN, *LEU2*, P*ADH1*, T*ADH1,* N-terminal G4AD-HA	this study
pG4BDC22-*RPS6A*	CEN, *TRP1*, P*ADH1*, T*ADH1,* C-terminal G4BD-c-Myc	this study
pG4BDC22-*RPS1B*	CEN, *TRP1*, P*ADH1*, T*ADH1,* C-terminal G4BD-c-Myc	this study
pG4BDC22-*RPS14A*	CEN, *TRP1*, P*ADH1*, T*ADH1,* C-terminal G4BD-c-Myc	this study

pGAG4BDC22-*RPL1B*	CEN, *TRP1*, P*ADH1*, T*ADH1,* C-terminal $(GA)_5$-G4BD-c-Myc	this study
pGAG4BDC22-*RPL18A*	CEN, *TRP1*, P*ADH1*, T*ADH1,* C-terminal $(GA)_5$-G4BD-c-Myc	this study
pGAG4BDC22-*RPL41B*	CEN, *TRP1*, P*ADH1*, T*ADH1,* C-terminal $(GA)_5$-G4BD-c-Myc	this study
pG4BDN22-*RPL39*	CEN, *TRP1*, P*ADH1*, T*ADH1,* N-terminal $(GA)_5$-G4BD-c-Myc	this study
pGAG4BDC22-*RPL42A*	CEN, *TRP1*, P*ADH1*, T*ADH1,* C-terminal $(GA)_5$-G4BD-c-Myc	this study
pGAG4ADC111-*RPL42A*	CEN, *LEU2*, P*ADH1*, T*ADH1,* C-terminal $(GA)_5$-G4AD-HA	this study
pG4BDC22-*RPS6A (1-123)*	CEN, *TRP1*, P*ADH1*, T*ADH1,* C-terminal G4BD-c-Myc	this study
pG4BDC22-*RPS6A (117-236)*	CEN, *TRP1*, P*ADH1*, T*ADH1,* C-terminal G4BD-c-Myc	this study
pG4BDC22-*RPS6A (117-181)*	CEN, *TRP1*, P*ADH1*, T*ADH1,* C-terminal G4BD-c-Myc	this study
pG4BDC22-*RPS6A (1-181)*	CEN, *TRP1*, P*ADH1*, T*ADH1,* C-terminal G4BD-c-Myc	this study
pG4BDC22-*RPS6A (176-236)*	CEN, *TRP1*, P*ADH1*, T*ADH1,* C-terminal G4BD-c-Myc	this study
pGAG4ADC111-*NAP1 (75-417)*	CEN, *LEU2*, P*ADH1*, T*ADH1,* C-terminal $(GA)_5$-G4AD-HA	this study
pG4ADHAN111-*NAP1 (75-417)*	CEN, *LEU2*, P*ADH1*, T*ADH1,* N-terminal G4AD-HA	this study
pGAG4ADC111-*NAP1 (1-365)*	CEN, *LEU2*, P*ADH1*, T*ADH1,* C-terminal $(GA)_5$-G4AD-HA	this study
pG4ADHAN111-*NAP1 (1-365)*	CEN, *LEU2*, P*ADH1*, T*ADH1,* N-terminal G4AD-HA	this study
pG4BDN22-*NAP1*	CEN, *TRP1*, P*ADH1*, T*ADH1,* N-terminal G4BD-c-Myc	this study
pGAG4BDC22-*NAP1*	CEN, *TRP1*, P*ADH1*, T*ADH1,* C-terminal $(GA)_5$-G4BD-c-Myc	this study
pG4BDN22-*NAP1 (75-417)*	CEN, *TRP1*, P*ADH1*, T*ADH1,* N-terminal G4BD-c-Myc	this study
pGAG4BDC22-*NAP1 (75-417)*	CEN, *TRP1*, P*ADH1*, T*ADH1,* C-terminal $(GA)_5$-G4BD-c-Myc	this study
pG4BDN22-*NAP1 (1-365)*	CEN, *TRP1*, P*ADH1*, T*ADH1,* N-terminal G4BD-c-Myc	this study
pGAG4BDC22-*NAP1 (1-365)*	CEN, *TRP1*, P*ADH1*, T*ADH1,* C-terminal $(GA)_5$-G4BD-c-Myc	this study
pADH195-*RPS6A*	2μ, *URA3*, P*ADH1*, T*ADH1*	this study
pG4BDC22-*RPS2 (1-22)*	CEN, *TRP1*, P*ADH1*, T*ADH1,* C-terminal G4BD-c-Myc	this study
pG4BDC22-*RPS2 (1-42)*	CEN, *TRP1*, P*ADH1*, T*ADH1,* C-terminal G4BD-c-Myc	this study
pG4BDC22-*RPS2* ΔN22 *(23-254)*	CEN, *TRP1*, P*ADH1*, T*ADH1,* C-terminal G4BD-c-Myc	this study
pG4BDC22-*RPS2* ΔN42 *(43-254)*	CEN, *TRP1*, P*ADH1*, T*ADH1,* C-terminal G4BD-c-Myc	this study
pG4ADC111-*TSR4* ΔN19 *(20-409)*	CEN, *LEU2*, P*ADH1*, T*ADH1,* C-terminal G4AD-HA	this study

pG4ADC111-*TSR4* ΔN40 *(41-409)*	CEN, *LEU2*, P*ADH1*, T*ADH1,* C-terminal G4AD-HA	this study
pG4ADC111-*TSR4 (1-400)*	CEN, *LEU2*, P*ADH1*, T*ADH1,* C-terminal G4AD-HA	this study
pRS316-*TSR4*	CEN, *URA3,* P*TSR4,* T*TSR4*	this study
pRS315-*TSR4*	CEN, *LEU2*, P*TSR4,* T*TSR4*	this study
pRS316-*RPS2*	CEN, *URA3*, P*RPS2,* T*RPS2*	this study
pRS315-*RPS2*	CEN, *LEU2*, P*RPS2,* T*RPS2*	this study
pRS314-*RPS2*	CEN, *TRP1*, P*RPS2,* T*RPS2*	this study
pRS314-*rps2* ΔN22 *(23-254)*	CEN, *TRP1*, P*RPS2,* T*RPS2*	this study
pRS314- *rps2* ΔN42 *(43-254)*	CEN, *TRP1*, P*RPS2,* T*RPS2*	this study
pRS314-*rps2-1*	CEN, *TRP1*, P*RPS2,* T*RPS2*	this study
pRS314-*rps2-2*	CEN, *TRP1*, P*RPS2,* T*RPS2*	this study
pRS315-*tsr4-1*	CEN, *LEU2*, P*TSR4,* T*TSR4*	this study
pRS315-*tsr4-2*	CEN, *LEU2*, P*TSR4,* T*TSR4*	this study
pADH111-*RPS2*-$(GA)_5$-3xyEGFP	CEN, *LEU2*, P*ADH1*, T*ADH1*, C-terminal $(GA)_5$-3xyEGFP	this study
pFA6a-HIS3MX4	for chromosomal deletion	(6)
pFA6a-kanMX4	for chromosomal deletion	(6)
pFA6a-TAP-HIS3MX4	TAP, T*ADH1*; for genomic C-terminal tagging	(4)
pFA6a-TAP-TCYC1-natNT2	TAP, T*CYC1*; for genomic C-terminal tagging	(7)
pFA6a-Flag-natNT2	1xFlag, T*CYC1*; for genomic C-terminal tagging	(8)
pETDuet-1-Flag-Rps6	Amp^r, T7 promoter/*lac* operator; Flag-Rps6 in MCS2	this study
pETDuet-1-His6-Nap1	Amp^r, T7 promoter/*lac* operator; Nap1 in MCS1	this study
pETDuet-1-His6-Nap1/Flag-Rps6	Amp^r, T7 promoter/*lac* operator; Nap1 in MCS1, Flag-Rps6 in MCS2	this study
pETDuet-1-Rps2-His6	Amp^r, T7 promoter/*lac* operator; Rps2-His6 in MCS1	this study
pCOLADuet-1-Tsr4-Flag	Kan^r, T7 promoter/*lac* operator; Tsr4-Flag in MCS2	this study

Supplementary Figure 1

A.

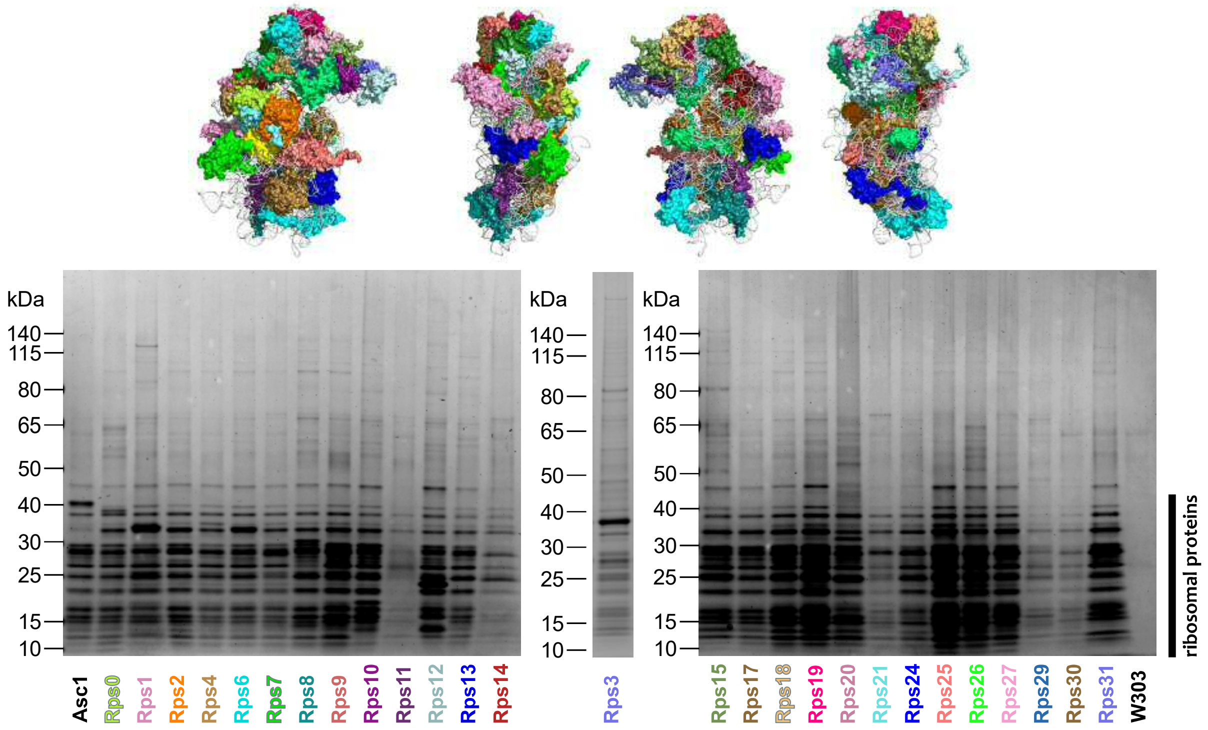

B.

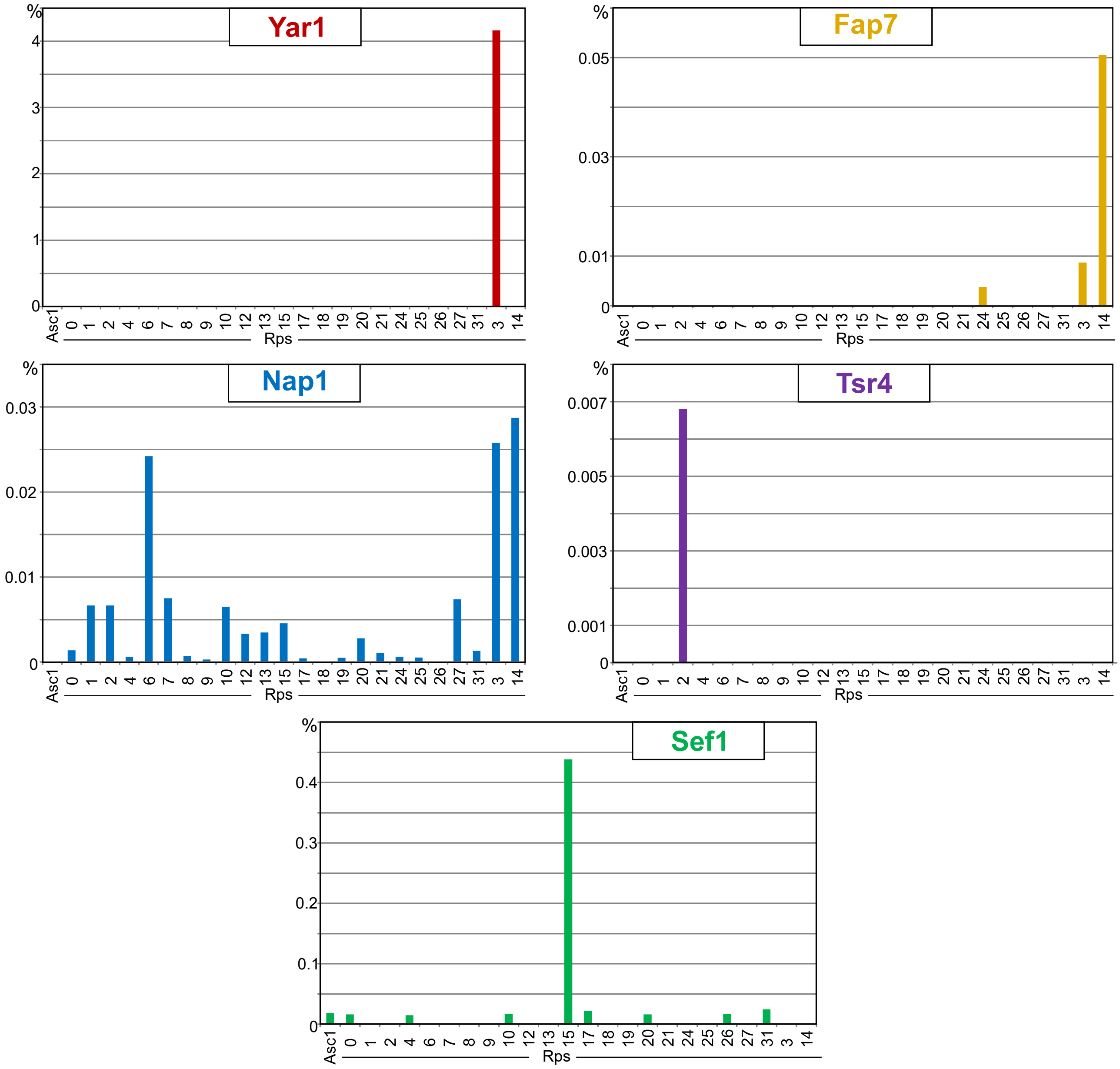

Supplementary Figure 1:

A. Coomassie-stained SDS-PAGE showing eluates after tandem-affinity purification of chromosomally C-terminally TAP-tagged ribosomal proteins of the small 40S subunit. For duplicated r-proteins (A and B), only one copy was tagged (listed in Supplementary Table 1). On top, the structure of the 40S subunit (PDB 4V88) is shown to indicate the positions of the respectively colored r-proteins. **B.** Relative intensities of known and putative dedicated chaperones in all 40S r-protein purifications.

Supplementary Figure 2

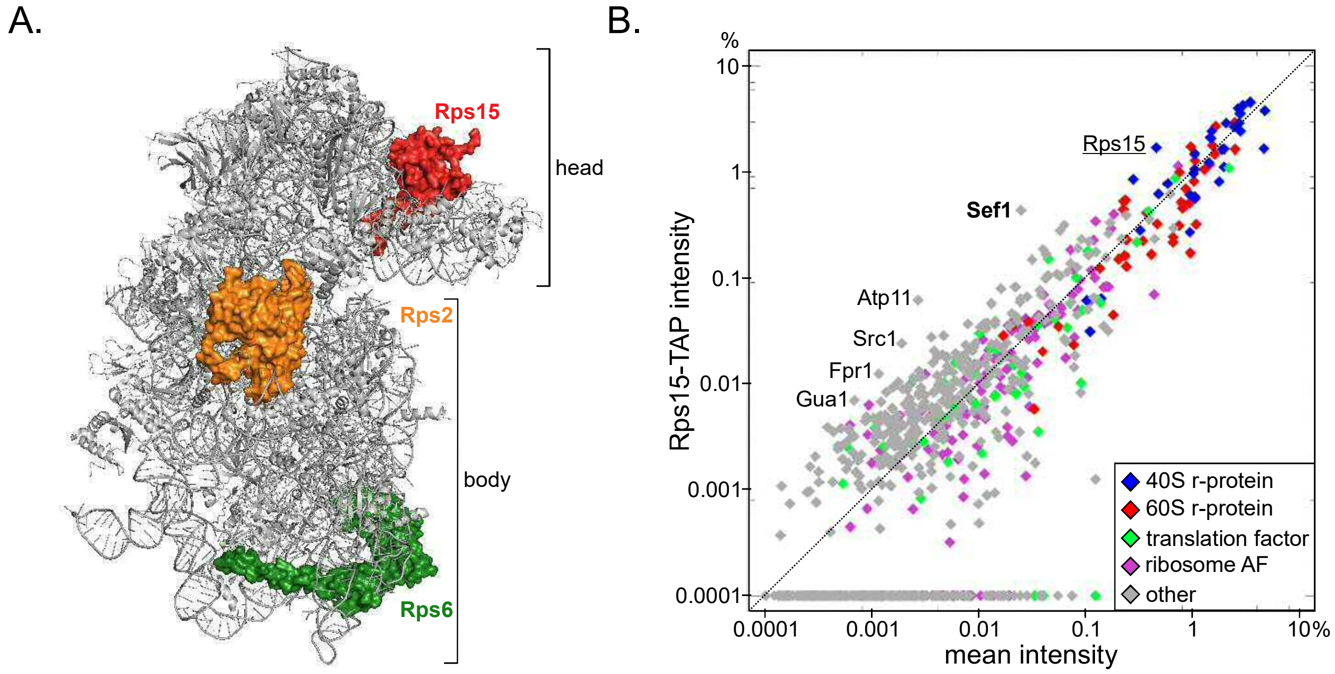

Supplementary Figure 2:

A. Positioning of Rps2, Rps6, and Rps15 in the 40S subunit. 40S structure (PDB 4V88) with the main structural domains of the 40S subunit and Rps2, Rps6, and Rps15 indicated in colors and surface representation. **B**. Relative intensities in the Rps15-TAP purification plotted against the mean intensity of all purifications. The Rps15-bait protein (underlined) and proteins particularly enriched in the purification are labeled, with Sef1, considered as potential dedicated chaperone candidate, indicated in bold.

Supplementary Figure 3

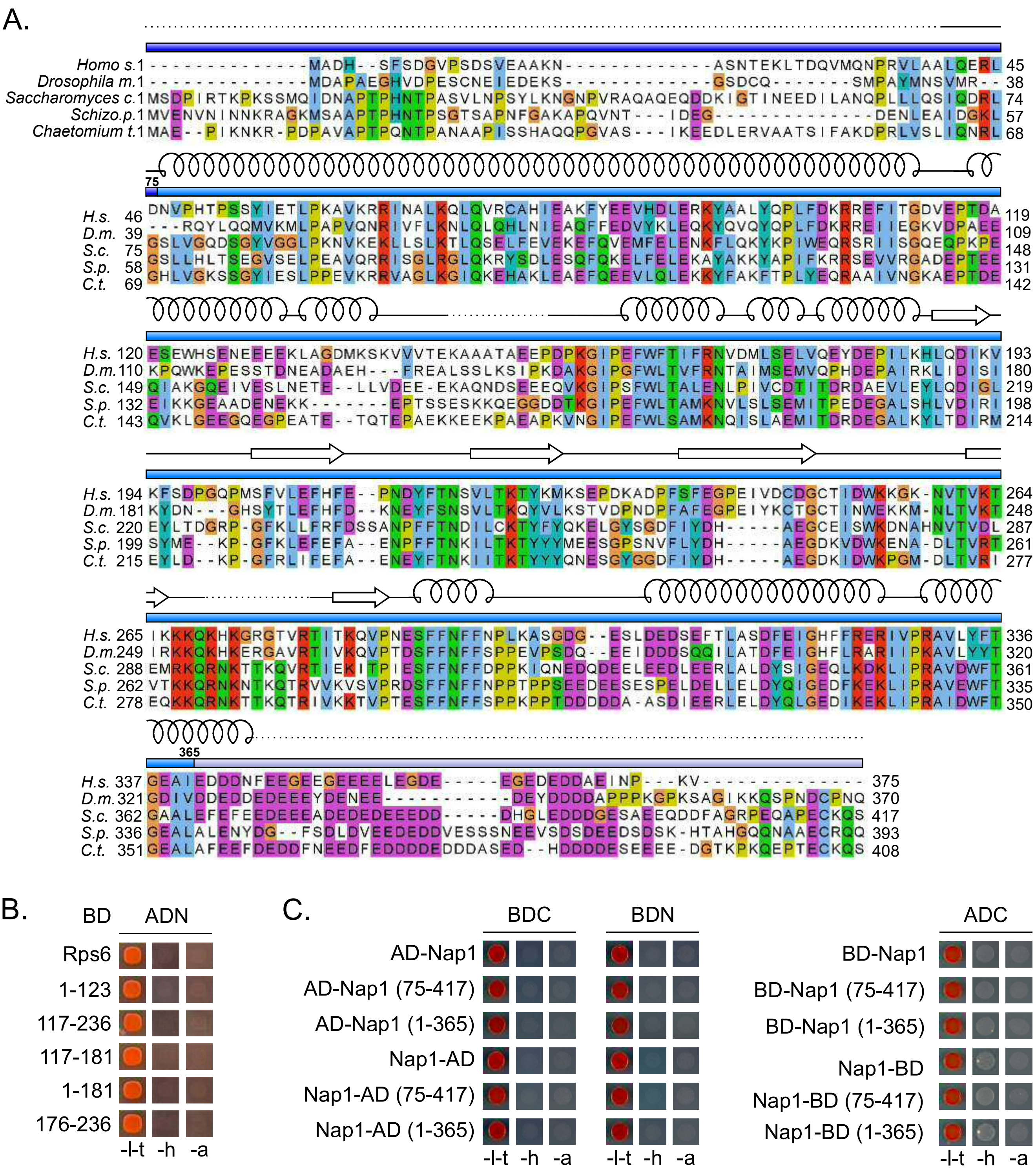

Supplementary Figure 3:
A. Sequences of Nap1, which is only present in eukaryotes, were aligned with Clustal Omega and viewed in Jalview. Truncations of Nap1 used in this study are indicated above the sequence in according colors. **B and C.** Y2H negative controls for the experiments shown in Figures 3B **(B.)**, 3C, and 3D **(C.)**. –l–t, SDC –leucine –tryptophan; –h, SDC –leucine –tryptophan –histidine; –a, SCD –leucine –tryptophan –adenine.

Supplementary Figure 4

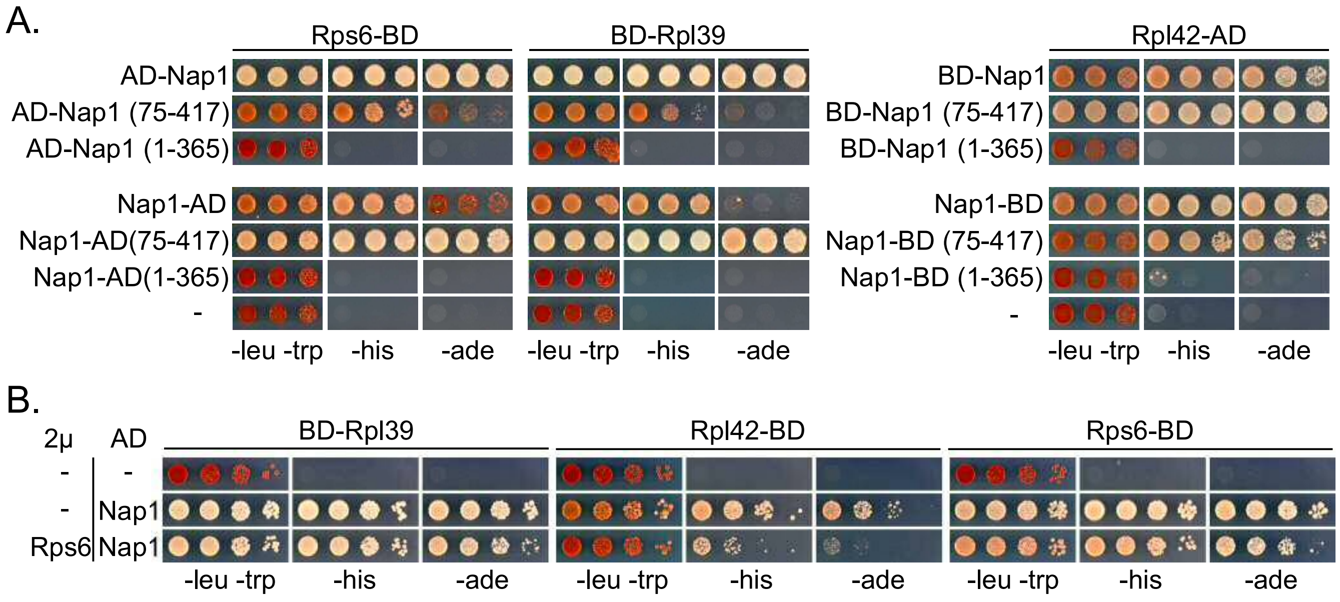

Supplementary Figure 4:

A. Binding of truncated Nap1 variants to Rps6, Rpl39, and Rpl42. Note that, depending on the construct, the N- and sometimes the C-terminal fusions of Nap1 fragments show stronger interaction, suggesting steric hindrance by the bulky AD or BD fusions in some cases. Combinations showing the strongest interaction (likely representing the respective situations with the least steric hindrance) are shown in Figure 3C and 3D. **B**. Expression of Rps6 from a multicopy plasmid reduces the Y2H interaction of Nap1 with Rpl39 and Rpl42. Combinations of Nap1 N-terminal Gal4 activation domain (AD) fusions with the indicated r-protein Gal4 DNA-binding domain (BD) fusions were transformed into PJ69-4A containing either a high-copy (2μ) *URA3*-plasmid expressing Rps6 or an empty plasmid. Cells were spotted on SDC –leucine –tryptophan –uracil plates (labeled –leu –trp), SDC –leucine –tryptophan –uracil –histidine plates (labeled –his), and SDC –leucine –tryptophan –uracil –adenine plates (labeled –ade). Note that in the presence of the Rps6-expressing plasmid, the Y2H interaction of Nap1 with Rpl39 and Rpl42, but also the Y2H interaction with Rps6, was reduced, as obvious from the smaller colonies on –ade plates, indicating competition between these Nap1-binding partners.

Supplementary Figure 5

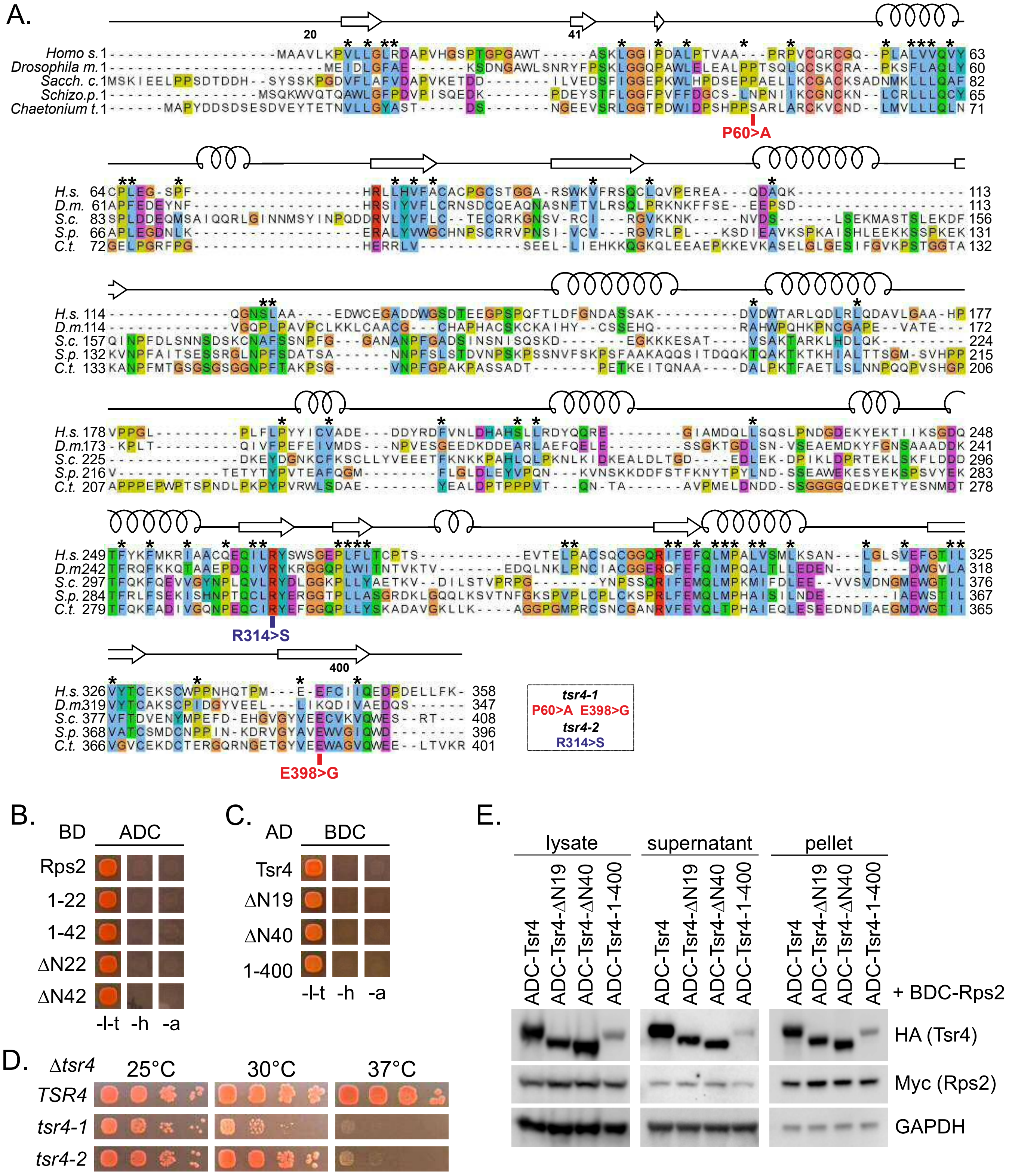

Supplementary Figure 5:

A. Sequences of Tsr4, which is only present in eukaryotes, were aligned with Clustal Omega, and viewed in Jalview. A secondary structure prediction based on the same alignment was performed in JPred and predicted α-helices and β-strands are indicated. Conserved hydrophobic amino acids are labeled by an asterisk. Mutations introduced into Tsr4 are marked in the sequence and listed on the bottom right. **B. and C.** Y2H interaction assays of the negative controls for Figure 5B and 5C. –l–t, SDC –leucine –tryptophan; –h, SDC –leucine –tryptophan –histidine; –a, SDC –leucine –tryptophan –adenine. **D.** Growth phenotype of *tsr4* point mutants. *TSR4* knockout cells carrying *TSR4*, *tsr4-1* or *tsr4-2* on a *LEU2* plasmid, were spotted on SDC –leucine plates and incubated at indicated temperatures for 3 days. Note that the growth defect of *tsr4-1* was stronger as compared to the Δ*rps2* Δ*tsr4* strain in which *tsr4-1* and *RPS2* were provided on plasmids (shown in Figure 6E). We speculate that the increased *RPS2* copy number in the experiment in Figure 6E compensated partially for the defects of the *TSR4* mutations. **E.** Expression and solubility test for the Y2H combinations tested in Figure 5C. Cells were lysed, followed by centrifugation of the lysate at 18,000x g to pellet insoluble material. The lysate, soluble proteins (supernatant), and insoluble proteins (pellet) were subsequently analyzed by Western blotting. The Gal4 activation domain (AD) fusion constructs were detected via their HA-tag, whereas the Gal4 DNA-binding domain (BD) fusion constructs were detected via their c-Myc-tag. Glyceraldehyde-3-phosphate-dehydrogenase (GAPDH) served as loading control.

Supplementary Figure 6

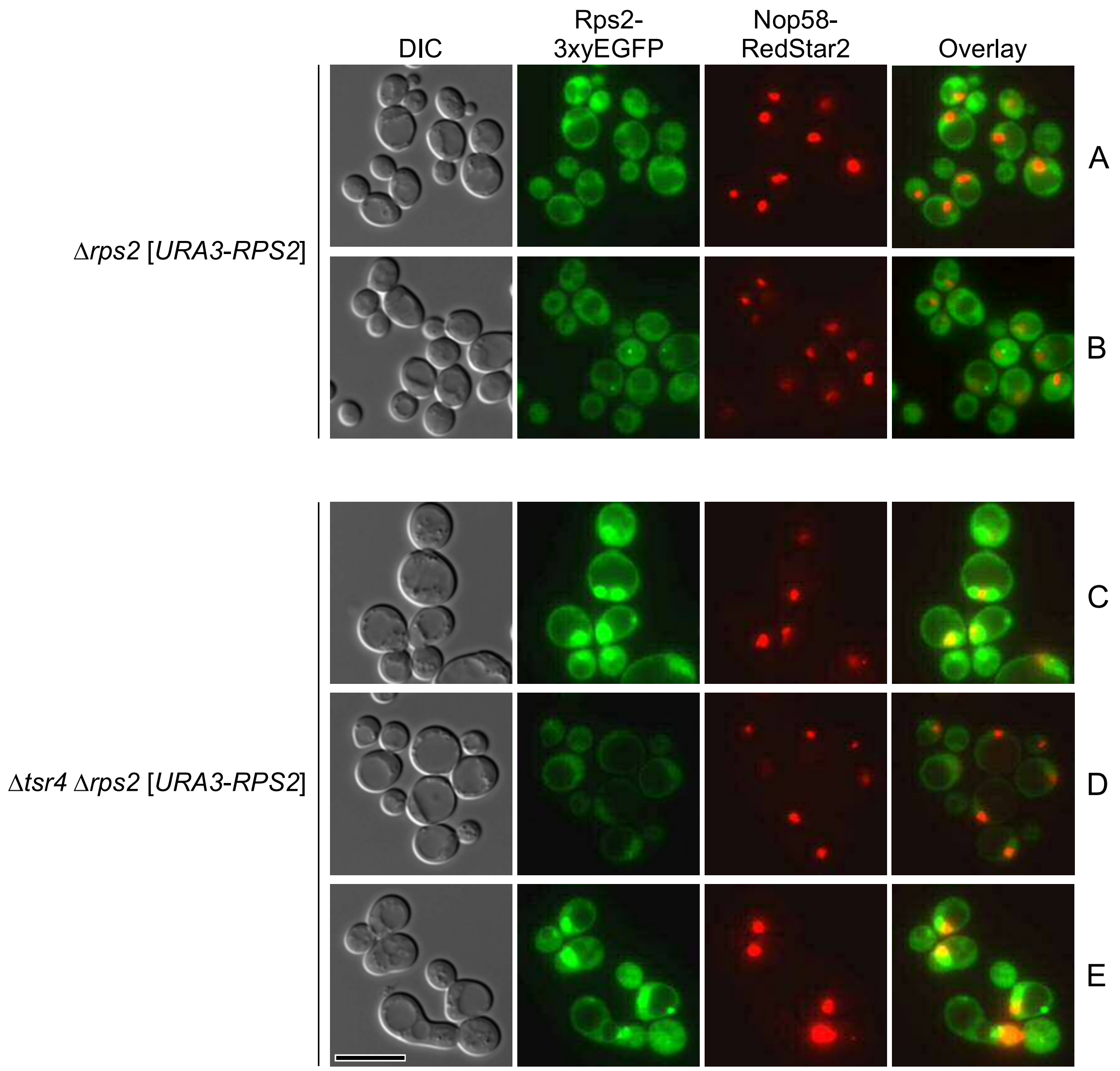

Supplementary Figure 6:
Fluorescence microscopy of Δ*rps2* and Δ*rps2* Δ*tsr4* strains, containing both a *URA3-RPS2* and a *LEU2-RPS2*-3xyEGFP plasmid, and a chromosomal C-terminal RedStar2-fusion of the nucleolar marker Nop58. The scale bar in the lower left panel represents 10 µm. All pictures were equally processed. Panels A, C, and D represent the same panels as shown in Figure 6C. In panel B, cells with small Rps2-GFP aggregates, which are occasionally occurring in wild-type cells, are shown. Panel E highlights the observation that Δ*tsr4* cells show abnormal morphologies, including budding defects.

Supplementary References

1. Thomas,B.J. and Rothstein,R. (1989) Elevated recombination rates in transcriptionally active DNA. *Cell*, **56**, 619–630.

2. Koch,B., Mitterer,V., Niederhauser,J., Stanborough,T., Murat,G., Rechberger,G., Bergler,H., Kressler,D. and Pertschy,B. (2012) Yar1 protects the ribosomal protein Rps3 from aggregation. *J. Biol. Chem.*, **287**, 21806–21815.

3. James,P., Halladay,J. and Craig,E.A. (1996) Genomic libraries and a host strain designed for highly efficient two-hybrid selection in yeast. *Genetics*, **144**, 1425–1436.

4. Pausch,P., Singh,U., Ahmed,Y.L., Pillet,B., Murat,G., Altegoer,F., Stier,G., Thoms,M., Hurt,E., Sinning,I., *et al.* (2015) Co-translational capturing of nascent ribosomal proteins by their dedicated chaperones. *Nat Commun*, **6**, 7494.

5. Mitterer,V., Murat,G., Réty,S., Blaud,M., Delbos,L., Stanborough,T., Bergler,H., Leulliot,N., Kressler,D. and Pertschy,B. (2016) Sequential domain assembly of ribosomal protein S3 drives 40S subunit maturation. *Nat Commun*, **7**, 10336.

6. Longtine,M.S., McKenzie,A., Demarini,D.J., Shah,N.G., Wach,A., Brachat,A., Philippsen,P. and Pringle,J.R. (1998) Additional modules for versatile and economical PCR-based gene deletion and modification in Saccharomyces cerevisiae. *Yeast*, **14**, 953–961.

7. Kressler,D., Roser,D., Pertschy,B. and Hurt,E. (2008) The AAA ATPase Rix7 powers progression of ribosome biogenesis by stripping Nsa1 from pre-60S particles. *J. Cell Biol.*, **181**, 935–944.

8. Kater,L., Thoms,M., Barrio-Garcia,C., Cheng,J., Ismail,S., Ahmed,Y.L., Bange,G., Kressler,D., Berninghausen,O., Sinning,I., *et al.* (2017) Visualizing the Assembly Pathway of Nucleolar Pre-60S Ribosomes. *Cell*, **171**, 1599-1610.e14.
